# Akt Regulates TNFα Synthesis Downstream of RIP1 Kinase Activation during Necroptosis

**DOI:** 10.1371/journal.pone.0056576

**Published:** 2013-03-01

**Authors:** Colleen R. McNamara, Ruchita Ahuja, Awo D. Osafo-Addo, Douglas Barrows, Arminja Kettenbach, Igor Skidan, Xin Teng, Gregory D. Cuny, Scott Gerber, Alexei Degterev

**Affiliations:** 1 Graduate Program in Biochemistry, Sackler School of Graduate Biomedical Sciences, Tufts University, Boston, Massachussets, United States of America; 2 Department of Biochemistry, Sackler School of Graduate Biomedical Sciences, Tufts University, Boston, Massachussets, United States of America; 3 Department of Genetics, Dartmouth Medical School, Lebanon, New Hampshire, United States of America; 4 Laboratory for Drug Discovery in Neurodegeneration, Harvard NeuroDiscovery Center, Brigham and Women’s Hospital and Harvard Medical School, Cambridge, Massachussets, United States of America; Juntendo University School of Medicine, Japan

## Abstract

Necroptosis is a regulated form of necrotic cell death that has been implicated in the pathogenesis of various diseases including intestinal inflammation and systemic inflammatory response syndrome (SIRS). In this work, we investigated the signaling mechanisms controlled by the necroptosis mediator receptor interacting protein-1 (RIP1) kinase. We show that Akt kinase activity is critical for necroptosis in L929 cells and plays a key role in TNFα production. During necroptosis, Akt is activated in a RIP1 dependent fashion through its phosphorylation on Thr308. In L929 cells, this activation requires independent signaling inputs from both growth factors and RIP1. Akt controls necroptosis through downstream targeting of mammalian Target of Rapamycin complex 1 (mTORC1). Akt activity, mediated in part through mTORC1, links RIP1 to JNK activation and autocrine production of TNFα. In other cell types, such as mouse lung fibroblasts and macrophages, Akt exhibited control over necroptosis-associated TNFα production without contributing to cell death. Overall, our results provide new insights into the mechanism of necroptosis and the role of Akt kinase in both cell death and inflammatory regulation.

## Introduction

Necroptosis is a form of regulated cell death that displays all the major hallmarks of necrosis [1]. A growing number of studies have implicated necroptosis in a wide range of animal models of human disease, including brain, heart and retinal ischemia-reperfusion injury, acute pancreatitis, brain trauma, retinal detachment, and Huntington’s disease [2], [3]. Importantly, several recent studies have linked necroptosis to models of inflammation including intestinal inflammation and systemic inflammatory response syndrome (SIRS) [4], [5], [6]. The discovery of a regulated form of necrotic death could uncover molecular targets amenable to pharmacological intervention for the treatment of various conditions.

A complex consisting of two related Ser/Thr kinases, RIP1 and RIP3, plays a critical role in the initiation of necroptosis in multiple systems [7], [8], [9]. A recent genome wide siRNA screen for mediators of necroptosis induced by the pan-caspase inhibitor zVAD.fmk in mouse fibrosarcoma L929 cells, revealed a broad and diverse cellular network of 432 genes that may regulate this process [10]. These data provided important confirmation of the highly regulated nature of necroptosis and revealed the first insight into the full repertoire of mediators of this form of cell death. However, the specific signaling pathways activated during necroptosis and their connections to RIP1 and RIP3 remain poorly understood. Several recent studies [10], [11], [12], [13], [14] have suggested that JNK kinase activation plays an important role during necroptosis in L929 cells downstream from RIP1 kinase. For example, the transcription factor c-Jun, a key cellular target of JNK activity, was one of the hits in the genome wide siRNA screen [10]. Activation of JNK in L929 cells has been linked to autocrine TNFα synthesis, activation of oxidative stress and induction of autophagy, all of which contribute to necroptosis. Importantly, RIP1 kinase dependent activation of JNK and TNFα production has recently been described to be independent of its role in necroptosis [15]. Curiously, Akt kinase, a key pro-survival molecule and a well-established inhibitor of apoptotic cell death, has also recently been linked to necroptosis in L929 cells [16], where insulin-dependent activation of Akt was suggested to promote necroptosis by suppressing autophagy. This conclusion was unexpected, since several reports from different groups, including ours, have established that autophagy promotes, rather than suppresses, zVAD.fmk-induced necroptosis in L929 cells [11], [14], [17]. This raised the possibility that Akt controls more general mechanisms that contribute to the execution of necroptosis. Furthermore, the key question of whether insulin-dependent Akt activity solely provides an environment conducive for necroptosis or if Akt activation is an intrinsic component of necroptosis signaling that is linked to RIP1 kinase has not been explored.

In this study, we expanded these observations to delineate the specific contributions and molecular ordering of the Akt and JNK pathways downstream from RIP1 kinase during necroptosis. Our data reveal that Akt is activated through RIP1 kinase-dependent Thr308 phosphorylation during necroptosis in multiple cell types. Furthermore, we found that downstream Akt signaling through mTORC1 and S6 contributes to the activation of necroptosis and TNFα production. We found that the Akt pathway serves as a critical link between RIP1 kinase and JNK activation in L929 cells. Further data suggested that in multiple other cell types including FADD deficient Jurkat cells, RAW and J774.1 macrophage cell lines, and mouse lung fibroblasts Akt provides a key link to TNFα production, but is dispensible for cell death *per se*. Overall, our results reveal a specific and novel role for the Akt pathway in regulated necrosis and necrosis-associated inflammatory signaling.

## Results

### Basic Fibroblast Growth Factor Promotes Necroptosis in L929 Cells

**Figure 1:**
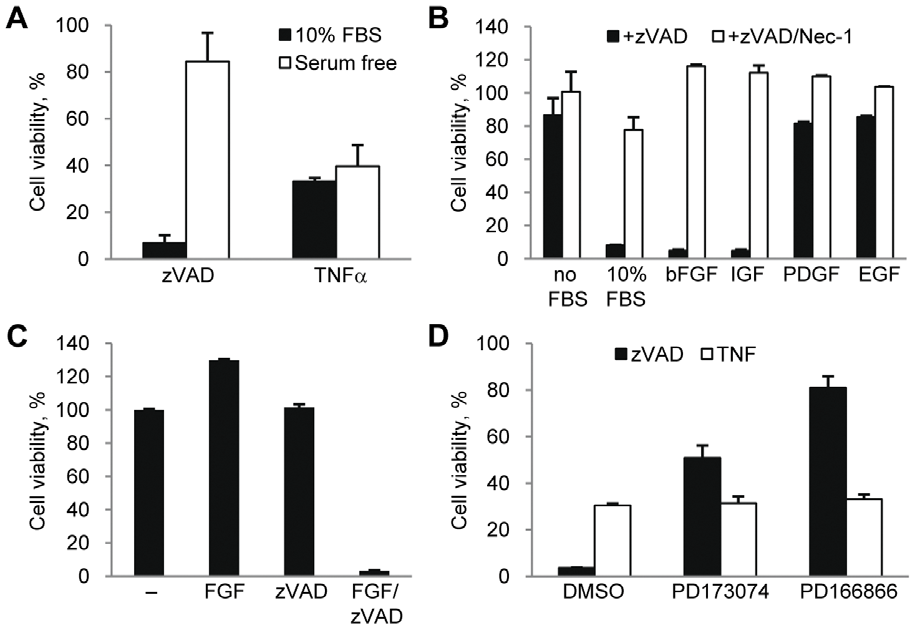
bFGF and IGF-1 promote necroptosis in concert with zVAD.fmk. (A) L929 cells were treated with TNFα or zVAD.fmk under normal serum (10% FBS) or serum free conditions. Cell viability was determined after 24 hr using the CellTiter-Glo Viability assay. The concentrations of all necroptosis-inducing agents are listed in the *Materials and Methods* section or indicated in the figures. (B) Cells were treated with zVAD.fmk, the indicated growth factors, and Nec-1 under serum free conditions for 24 hrs followed by measurement of cell viability. (C) Cells under serum free conditions were treated with FGF, zVAD.fmk, or both for 24 hrs followed by viability assay. (D) Cell death was induced by zVAD.fmk or TNFα under full serum condition in the presence of 2 µM PD173074 and 20 µM PD166866. In all graphs, average±SD was plotted.

It has been established that mouse fibrosarcoma L929 cells undergo necroptotic cell death following stimulation with TNFα [10], [17]. In addition, inhibition of caspase-8 activity alone, either through siRNA knockdown or by using the pan-caspase inhibitor, zVAD.fmk, is sufficient to trigger necroptosis in these cells [10], [14]. Interestingly, while necroptosis was initially identified as a back-up form of cell death triggered by pro-apoptotic stimuli in the presence of apoptosis inhibitors [17], recent analysis of physiological cell death during mouse development has suggested that the loss of apoptotic regulators, such as caspase-8 and FADD [18], [19], [20], leads to robust induction of necroptosis and death of E10.5 embryos even though apoptosis is not normally induced in wild type embryos. These data are reminiscent of the observations in L929 cells where the loss of caspase activity in healthy cells is sufficient to trigger necroptosis and prompted us to explore the extrinsic or intrinsic cellular factors that promote necroptosis once caspase-8 activity, which cleaves and inactivates RIP1 kinase and the RIP1 deubiquitinase CYLD [21], [22], is removed in L929 cells. Consistent with a previous report [16], we found that serum starvation of L929 cells prevented necroptosis in response to zVAD.fmk (Fig. 1A). The addition of growth factors, such as bFGF, restored zVAD.fmk induced death under serum free conditions (Fig. 1B). Interestingly, this does not reflect a generic requirement for growth factor signaling, as only some growth factors (bFGF and IGF-1, but not EGF and PDGF) promoted death (Fig. 1B). Furthermore, growth factor-dependent necroptosis required the inhibition of caspase activity, as bFGF alone did not induce cell death (Fig. 1C). In contrast, TNFα triggered necroptosis equally efficiently in the absence of serum (Fig. 1A), suggesting that either growth factors and zVAD.fmk or TNFα are required for necroptotic death in L929 cells.

Previously we described the development of 7-Cl-O-Nec-1 (Nec-1) as a potent and selective inhibitor of RIP1 kinase and necroptosis (Fig. S1A) [23], [24]. Recently, its selectivity has been further validated against a panel of more than 400 human kinases [15]. This inhibitor efficiently blocked growth factor/zVAD.fmk-induced necroptosis under serum free conditions in L929 cells and both zVAD.fmk and TNFα-induced necroptosis under full serum conditions (Fig. 1B, S1B). To further validate the role of RIP1, we used an inactive analog, 7-Cl-O-Nec-1i (Nec-1i) (Fig. S1A), which contains an extra N-methyl group that leads to almost complete loss of RIP1 kinase inhibitory activity *in vitro* [23]. Nec-1i was unable to protect L929 cell death under serum condtions treated with zVAD.fmk or TNFα (Fig. S1B) or serum free conditions treated with bFGF/zVAD.fmk (Fig. S1C). This confirms that RIP1 kinase is responsible for necroptosis in L929 cells under both serum and serum free conditions.

We next examined whether bFGF contributes to zVAD.fmk-induced necroptosis under normal serum conditions (10% FBS). We used two bFGF receptor tyrosine kinase inhibitors (PD173074 and PD166866), and determined that inhibition of bFGF signaling strongly inhibited zVAD.fmk-induced necroptosis under normal serum conditions (Fig. 1D). In contrast, neither bFGF receptor inhibitor was able to attenuate TNFα-induced necroptosis (Fig. 1D), consistent with growth factors being dispensable for this pathway (Fig. 1A). Overall, these data suggest that the induction of necroptosis by zVAD.fmk is promoted by bFGF under both serum and serum free conditions. The induction of necroptosis, however, is not a simple consequence of growth factor signaling since not all growth factors allowed death to occur. Instead, specific signaling events mediated by particular growth factors appear to contribute to necroptotic death.

### RIP1 Kinase-dependent Activation of Akt Contributes to Necroptosis

**Figure 2:**
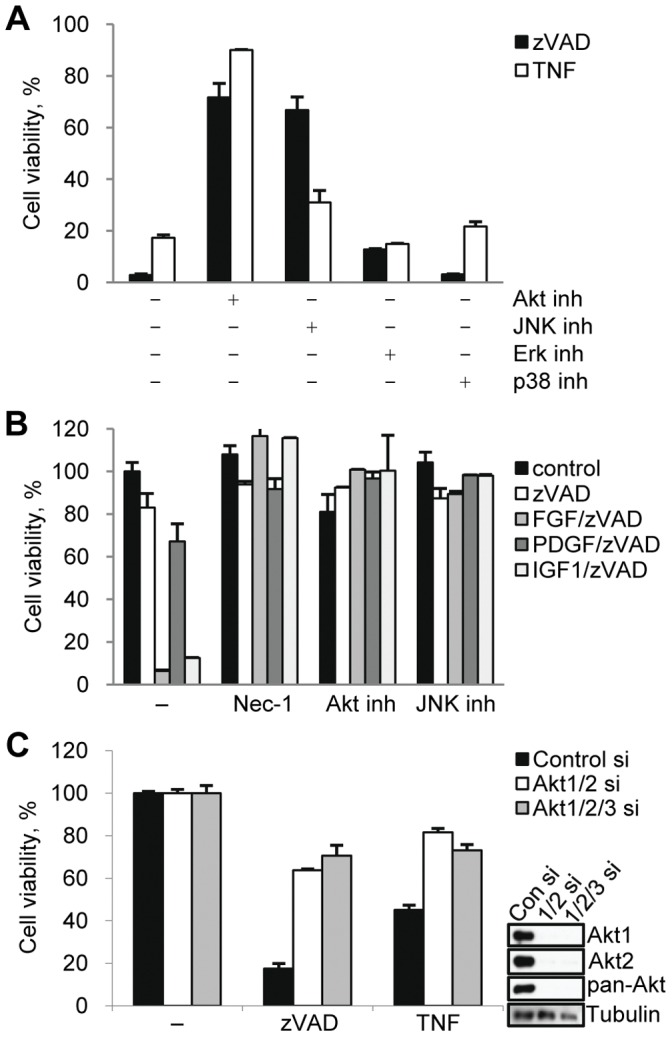
Akt contributes to necroptosis induced by zVAD.fmk and TNFα. (A,B) Necroptosis was induced by zVAD.fmk or TNFα (full serum, A) or growth factors/zVAD.fmk (serum free, B) in the presence of inhibitors of Akt (Akt inhibitor VIII), JNK (SP600125), p38 (PD169316), and Erk (UO126). Cell viability was determined after 24 hrs. (C) L929 cells transfected with Akt1, Akt2, and Akt3 siRNAs for 72 hrs were treated with zVAD.fmk or TNFα for 9 hrs. Cell viability and Akt expression levels were determined after 24 hrs. In all graphs, average±SD was plotted.

Given our observation that growth factors are important for zVAD.fmk induced death, we examined the contribution of several pathways, including MAPK pathways and Akt, which are known to be activated following growth factor receptor activation (Fig. 2A). Inhibition of Akt (Akt inhibitor VIII) strongly protected the cells from growth factor-sensitive necroptosis induced by zVAD.fmk [16] as well as cell death triggered by bFGF or IGF-1/zVAD.fmk under serum free conditions (Fig. 2B). Inhibition of Akt also protected the cells from growth-factor insensitive death by caused by TNF**α** (Fig. 2A). Consistent with previous reports, the JNK inhibitor SP600125 protected the cells from both zVAD.fmk and TNF**α** induced death (Fig. 2A,B and Fig. S2A) [12], [14]. In contrast, inhibition of two other MAPKs, p38 and ERK, previously reported not to be activated during necroptosis [14], did not protect from either zVAD.fmk or TNF**α** induced death (Fig. 2A).

Next, we used two approaches to further validate the role of Akt in necroptotic cell death. First, two additional Akt inhibitors, a highly specific, allosteric kinase inhibitor MK-2206 [25] and triciribine (TCN) [26], which blocks membrane translocation of Akt, both attenuated cell death (Fig. S2B). Secondly, simultaneous knockdown of Akt isoforms Akt1 and Akt2 using siRNAs protected cells from necroptosis induced by both zVAD.fmk and TNFα (Fig. 2C). No expression of Akt3 was seen in L929 cells (Fig. S2C) and, consistently, Akt3 siRNA had no additional effect on necroptosis. Our results confirmed that Akt plays a key role in necroptosis induced by multiple stimuli in L929 cells.

**Figure 3:**
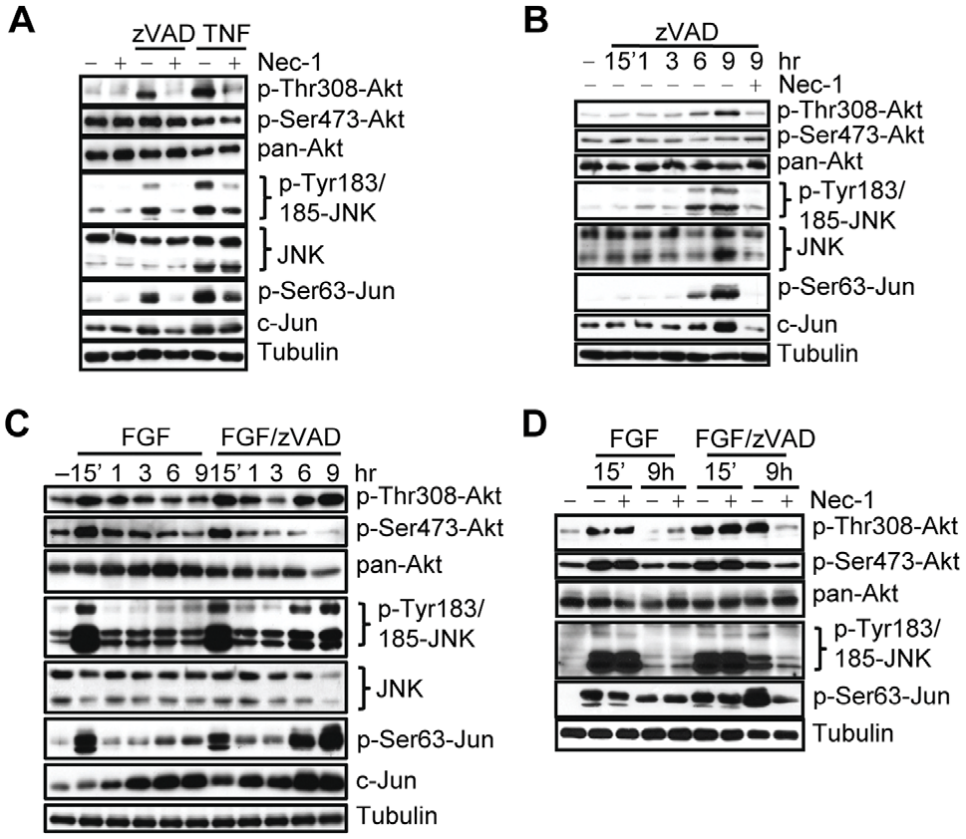
RIP1 kinase-dependent phosphorylation of Akt and JNK during necroptosis. (A) L929 cells were treated with zVAD.fmk or TNFα for 9 hr, followed by western blotting with indicated antibodies. (B,C) L929 cells were treated with zVAD.fmk (B) or bFGF/zVAD.fmk (serum free conditions, C) and samples were collected at the indicated time points for western blot. (D) Nec-1 was added to the cells stimulated with bFGF or bFGF/zVAD (serum free conditions) for 15 min or 9 hr followed by western blot with the indicated antibodies.

To understand the activation of Akt and JNK under necroptotic conditions, we examined the changes in Akt and JNK phosphorylation at 9 hrs post zVAD.fmk and TNFα stimulation. This time point was chosen because it reflects the early stage of cell death in our system (Fig. S3A, B). Following stimulation with either zVAD.fmk or TNFα we observed a robust increase in Akt phosphorylation at a known major activation site, Thr308 (Fig. 3A). Interestingly, we did not observe concomitant phosphorylation changes in the second major activation site of Akt, Ser473. We also observed an increase in the phosphorylation of both the p46 and p54 isoforms of JNK and its major substrate c-Jun (Fig. 3A). These data indicate that both Akt and JNK are activated under necroptotic conditions.

The RIP1 kinase inhibitor, Nec-1, completely prevented the increase in Thr308 Akt phosphorylation, while Nec-1i did not (Fig. 3A, Fig. S1D). Similarly, Nec-1 prevented the induction of JNK phosphorylation in response to zVAD.fmk and substantially reduced this change after TNFα addition. We observed some changes in total protein levels of JNK and c-Jun following necroptotic stimulation. Some of these changes, e.g. zVAD.fmk-induced increase in c-Jun, were also attenuated by Nec-1. Importantly, Nec-1 did not alter the basal phosphorylation levels of either Akt or JNK (Fig. 3A). This established that Akt Thr308 and JNK phosphorylation during necroptosis is RIP1 dependent.

Interestingly, we discovered that the phosphorylation of Akt Thr308, JNK and Jun are late events following zVAD.fmk stimulation (Fig. 3B) that coincide with the onset of necroptosis at 6 hr post-stimulation (Fig. S3A). To better understand the contributions of growth factors and RIP1 kinase to necroptotic regulation of Akt, we next analyzed the time course of these phosphorylation changes under serum free conditions. We found that the addition of bFGF alone or in combination with zVAD.fmk led to a substantial rapid and transient increase in both Thr308 and Ser473 phosphorylation of Akt as well as JNK and c-Jun at 15 minutes, reflecting the expected response to growth factor stimulation (Fig. 3C). Significantly, the combination of bFGF/zVAD.fmk, but not bFGF alone, also caused a robust, second, delayed increase in the phosphorylation of Thr308, but not Ser473, of Akt as well as a delayed increase in the phosphorylation of JNK and Jun. Furthermore, Nec-1 had no significant effect on the early increase in both Akt and JNK/c-Jun phosphorylation triggered by both bFGF and bFGF/zVAD, while Nec-1, but not its inactive analog Nec-1i (Fig. S1E), efficiently blocked the bFGF/zVAD increase at 6–9 hr (Fig. 3D), suggesting that only the delayed activation of Akt and JNK is specific for necroptosis and dependent on RIP1 kinase activity. Similarly, IGF/zVAD, which also promoted cell death under serum free conditions, produced a delayed increase in Thr308 phosphorylation on Akt, while IGF alone caused solely an early, transient increase in phosphorylation (Fig. S3C). We confirmed the kinetics of the Akt Thr308 and Ser473 phosphorylation changes using a quantitative ELISA assay, which also showed a robust delayed necroptosis-specific RIP1-dependent increase in Akt Thr308 phosphorylation (Fig. S3D, E). Taken together, these results indicate that the observed delayed increases in Akt and JNK phosphorylation, preceding the onset of cell death, represent specific consequences of necroptotic signaling downstream from RIP1 kinase.

### TNFα Induces Delayed Akt Thr308 Phosphorylation and Necroptosis Independent of Growth Factor Stimulation

Consistent with TNFα inducing necroptosis independently of growth factors (Fig. 1A), FGFR inhibitors did not attenuate TNFα-induced changes in Akt or JNK phosphorylation, while efficiently preventing these changes in response to zVAD.fmk (Fig. S4A). Furthermore, addition of TNFα led to comparable late activation of Akt p308 signal under both normal and serum free conditions (Fig. S4B, C), indicating that TNFα signaling to Akt Thr308 is growth factor-independent. In contrast, activation of JNK by TNFα followed different kinetics from zVAD.fmk-induced changes. TNFα treatment caused an early and robust increase in the phosphorylation of JNK and c-Jun. Nec-1 did not affect this early increase, however, it reduced levels of pJNK/Jun at the late, 9 hr time point (Fig. S4B, C). This again separated early RIP1-independent changes, which likely reflect the ability of additional upstream kinases, such as Ask1 to activate JNK [27], from the late RIP1 kinase-dependent necroptotic signaling.

### Late Increase in Akt Thr308 Phosphorylation Contributes to the Induction of Necroptotic Cell Death

**Figure 4:**
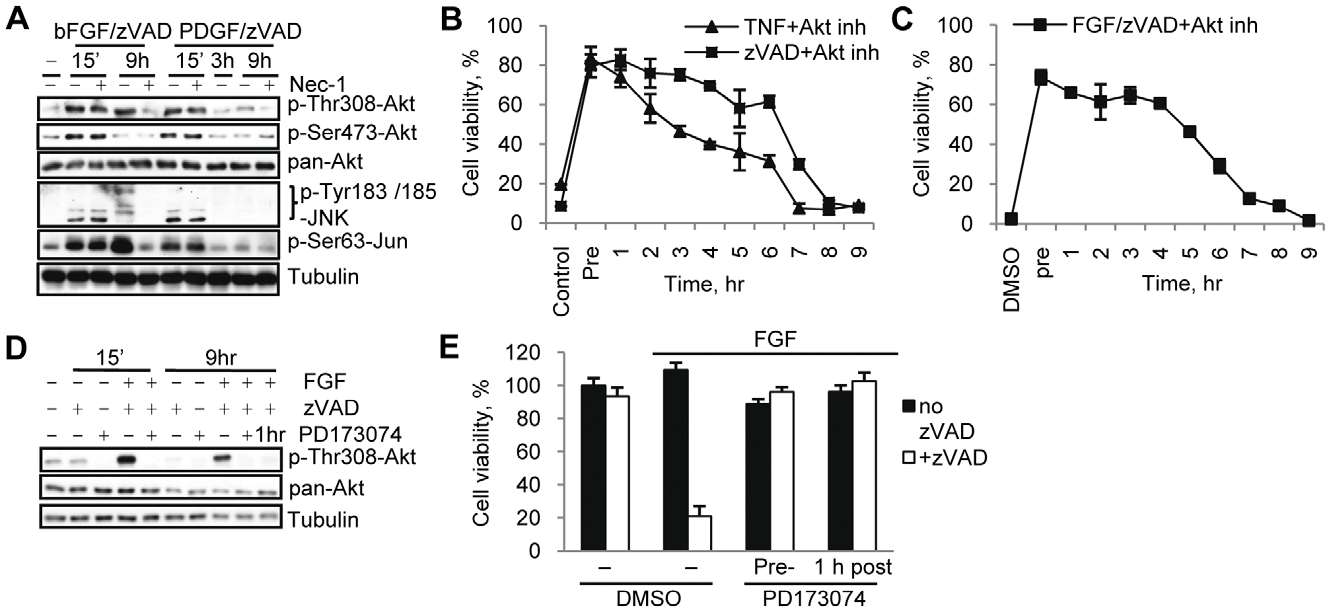
Late Thr308 phosphorylation of Akt contributes to necroptosis. (A) L929 cells were treated with zVAD.fmk and bFGF or PDGF, with or without Nec-1, for the indicated periods of time. (B,C) L929 cells were stimulated by zVAD.fmk or TNFα (B) or bFGF/zVAD.fmk under serum free conditions (C). Akt inh. VIII was added 15 min before necroptotic stimulation (Pre) or at indicated times after stimulation. Viability was measured 24 hr after activation of necroptosis. (D) L929 cells were stimulated with bFGF/zVAD under serum free conditions. PD173074 was added 15 min before or 1 hr after FGF/zVAD. Samples for western blot were collected at 15 min and 9 hr time points. (E) Cells were pretreated with PD173074 or it was added 1 hr after bFGF/zVAD.fmk, followed by viability assessment at 24 hr. In all graphs, average±SD was plotted.

We next investigated if the delayed RIP1 kinase-dependent increase in Akt Thr308 phosphorylation functionally contributes to the execution of necroptotic cell death. Firstly, PDGF/zVAD.fmk, which cannot induce necroptosis (Fig. 2A), triggered only the initial, rapid Akt and JNK phosphorylation changes and not the delayed activation (Fig. 4A), indicating that late, rather than early Akt phosphorylation correlates with necroptosis. Secondly, we saw that the ability of the Akt inhibitor to protect cells from necroptosis rapidly declined after 6 hrs of stimulation with zVAD.fmk, TNFα or bFGF/zVAD.fmk and no protection was observed when the inhibitor was added at 9 hrs (Fig. 4B,C). This time frame coincides with the timing of the secondary Akt Thr308 phosphorylation. Finally, we terminated the bFGF signal one hour after addition of bFGF by the addition of PD173074. This allowed us to retain early Akt activation, but to suppress the secondary increase (Fig. 4D). Both pre-addition and delayed addition of PD173074 fully prevented necroptosis (Fig. 4E). Overall, these data, while correlative, indicate that early Akt activation is insufficient to promote necroptosis and are strongly supportive of an important role for the delayed activation of Akt in the induction of necroptotic cell death.

### The Akt Signaling Pathway Contributes to the Regulation of Necroptosis

**Figure 5:**
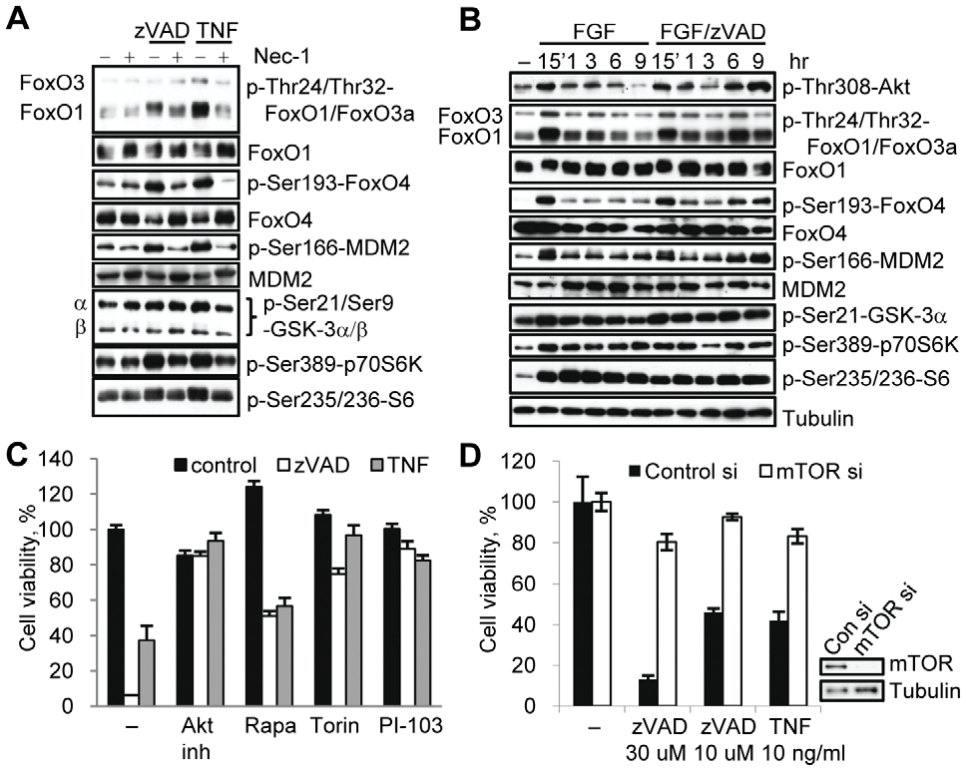
mTORC1 contributes to the regulation of necroptosis. (A) L929 cells were treated with zVAD.fmk or TNFα for 9 hr and harvested for western blot. (B) Cell under serum free condition were treated with bFGF or bFGF/zVAD.fmk for the indicated amounts of time, followed by western blotting using the indicated antibodies. (C) Necroptosis was induced by zVAD.fmk or TNFα in L929 cell in the presence of inhibitors of Akt(Akt inh. VIII) and mTOR (rapamycin, Torin-1 and PI-103). (D) L929 cells with mTOR siRNA knockdown were harvested for western blot or treated with zVAD.fmk or TNFα for 24 hrs. Cell viability was determined 24 hr after activation of necroptosis. In all graphs, average±SD was plotted.

We next determined whether the necroptosis-associated increase in Thr308 phosphorylation results in an increase in Akt kinase activity. Under necroptotic conditions, we observed an increase in the phosphorylation of multiple known Akt substrates (Forkhead box class O (FoxO) proteins, GSK-3 kinases and mouse double minute 2 (MDM2)) as well as downstream molecules (p70 ribosomal protein S6 Kinase (p70S6K), S6) (Fig. 5A). In some cases (FoxO1, FoxO4, MDM2), a robust increase was observed. In other cases (FoxO3a, GSK-3α/β, p70S6K and its substrate S6), the changes were less pronounced (Fig. 5A). The timing of the phosphorylation changes paralleled the increase in Akt phosphorylation (Fig. 5B, S5A, B). In the case of pFoxO1 we occasionally observed a shift in migration rather than an increase in band intensity (e.g. comparing Fig. 5A and B), suggesting that phosphorylation events in addition to Thr24 take place during necroptosis. Notably, in all cases the necroptosis-associated increases in Akt substrates were abrogated by Nec-1 (Fig. 5A, Fig. S5A, B). Overall, these data suggested that a significant part of the “canonical” Akt signaling network is activated at the onset of necroptotic cell death in a RIP1 dependent fashion.

Akt kinase is considered to be a pro-survival protein that inhibits apoptosis through the control of multiple effectors including mTORC1, GSK-3 and others [28]. An important question is whether these same molecules reverse their pro-survival roles during necroptosis. We found that inhibition of mTORC1 by rapamycin, an inhibitor of the mTOR co-factor Raptor [29], protected cells from necroptosis (Fig. 5C). Similarly, the direct mTOR kinase inhibitor Torin1 [30] and the dual PI3K/mTOR inhibitor PI-103 [31] also efficiently inhibited necroptosis (Fig. 5C). Knockdown of mTOR using siRNA further validated the small-molecule inhibitor data indicating a role for mTOR in necroptosis by protecting cells from both zVAD.fmk and TNFα induced death (Fig. 5D).

mTORC1 regulates translation through activation of p70S6 kinase and, subsequently, ribosomal protein S6 [32]. Notably, a genome-wide siRNA screen [10] suggested an important role for protein translation in necroptosis. Consistently, we found that the small molecule inhibitor of p70S6K PF-4708671 [33] attenuated necroptosis at the concentrations required to block S6 phosphorylation (Fig. S5C, D). Partial siRNA knockdown of S6 protein attenuated necroptosis as well (Fig. S5E), suggesting that translational control by p70S6K/S6 may play a role in necroptosis. Overall, while the full repertoire of Akt targets during necroptosis remains to be fully explored, our data provide evidence that the activity of an anti-apoptotic branch of Akt signaling can promote necroptosis.

**Figure 6:**
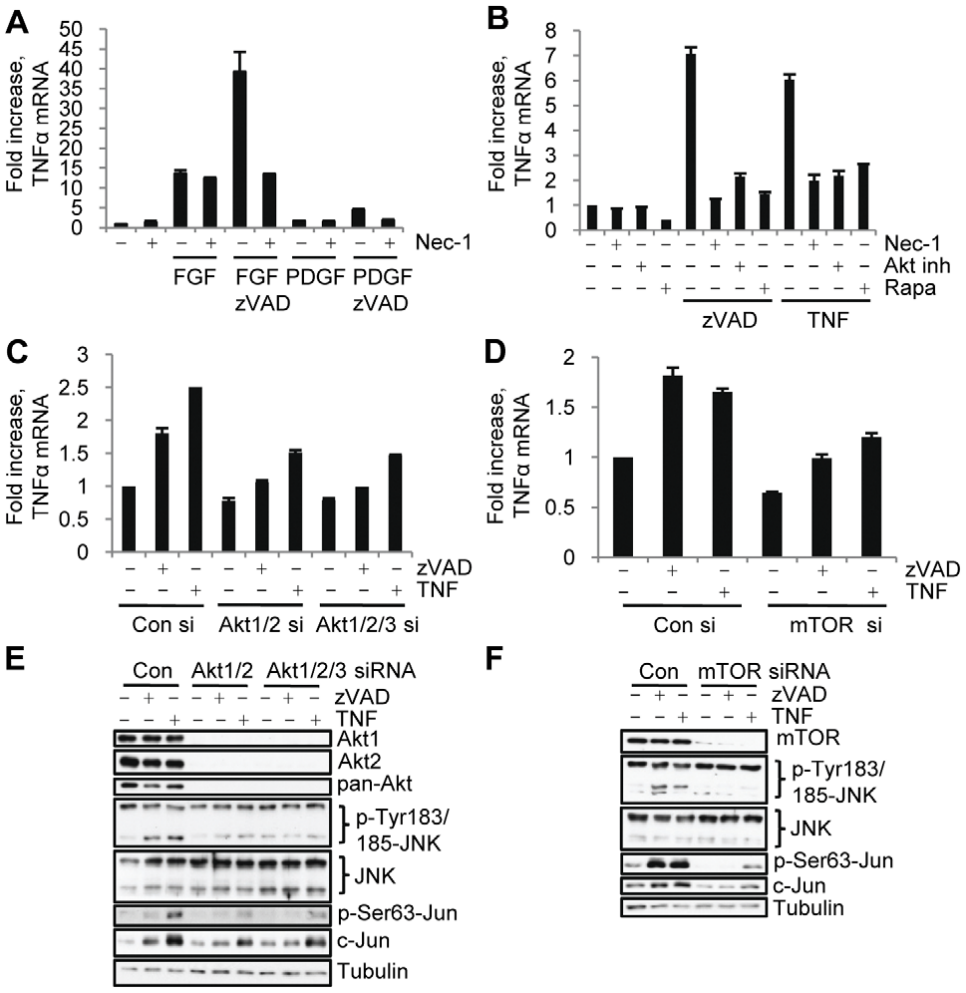
Akt and mTORC1 control autocrine TNFα synthesis and JNK activation during necroptosis. (A) Cells were treated under serum free conditions with bFGF or PDGF with or without zVAD.fmk for 9 hr, followed by qRT-PCR analysis of mTNFα. Data was normalized to mouse 18S RNA. (B) Necroptosis was induced by zVAD.fmk or TNFα in cells treated with Nec-1, rapamycin (rapa), or Akt inh. VIII inh. followed by qRT-PCR analysis of TNFα mRNA levels. (C-F) L929 cells with siRNA knockdown of Akt isoforms (C,E) or mTOR (D,F) were stimulated with zVAD.fmk or TNFα for 9 hr, followed by qRT-PCR analysis of mTNFα (C,D) or western blot (E,F). In all graphs, average±SD was plotted.

*RIP1 kinase, Akt, mTORC1 and JNK control the upregulation of TNF*α *accompanying necroptosis.* Hitomi et al. [10] have recently reported that the induction of necroptosis by zVAD.fmk in L929 cells is associated with increased synthesis of TNFα, which potentiates cell death. Therefore, we examined whether Akt and its effectors contribute to TNFα synthesis. Consistent with a RIP1-dependent increase in TNFα protein (Fig. S6A, B), we found that TNFα mRNA levels increased during necroptosis in L929 cells in a RIP1 (Fig. S6C. Under serum free conditions, bFGF alone triggered some induction of TNFα mRNA, while its combination with zVAD.fmk (but not zVAD.fmk alone) caused a pronounced further increase (Fig. 6A). Conversely, PDGF caused a modest upregulation of TNFα mRNA, which was not further increased in the presence of zVAD.fmk (Fig. 6A), demonstrating that activation of necroptosis is specifically accompanied by a marked increase in autocrine TNFα synthesis.

Further analysis suggested that both Akt and mTORC1 contribute to the upregulation of TNFα mRNA during necroptosis as both small-molecule inhibition and siRNA knockdown of Akt and mTOR reduced TNFα mRNA levels in necroptotic cells (Fig. 6B,C,D). Notably, RIP1 and Akt inhibitors had no effect on the levels of TNFα mRNA in control cells or in the cells stimulated with bFGF alone (Fig. 6A,B, Fig. S6C), suggesting that these kinases specifically mediate necroptosis-dependent increase in TNFα synthesis.

### Akt and mTORC1 Control the Activation of JNK during Necroptosis

JNK is a well-established regulator of TNFα synthesis in a variety of systems [13], [14], [15], [34]. Therefore, the ability of Akt and mTORC1 inhibitors to block the increase in TNFα mRNA lead us to examine their role in the activation of JNK during necroptosis. Knockdown of Akt isoforms Akt1 and Akt2 or inhibition of Akt prominently suppressed the necroptosis dependent increase in JNK and c-Jun phosphorylation (Fig. 6E, S6D,E) suggesting that Akt may provide a link between RIP1 and JNK activation. Importantly, inhibition of Akt only inhibited the delayed, but not the early, increase in bFGF/zVAD.fmk induced JNK and c-Jun phosphorylation (Fig. S6F). Knockdown of mTOR, rapamycin and the p70S6K inhibitor PF-4708671 also attenuated the necroptosis-associated increase in JNK and c-Jun phosphorylation (Fig. 6F, S6E,G, Fig. S5D). Overall, these data suggested that the Akt-mTORC1-S6K axis, acting downstream from RIP1 kinase, is required for the increase in JNK activity during necroptosis in L929 cells.

### PI3-kinase and PDK1 Mediate the Increase in Akt Thr308 Phosphorylation Under Necroptotic Conditions

Typical regulation of Akt by growth factors involves its recruitment to the plasma membrane, which is mediated by the binding of the pleckstrin homology (PH) domain of Akt to the product of PI3K, phosphatidylinositol-3,4,5-triphosphate (PIP3). In the membrane, Akt is phosphorylated on Thr308 and Ser473 by 3-phosphoinositide dependent protein kinase-1 (PDK1) and mTORC2 (or DNA-PK), respectively [35]. Since our results showed that only Thr308 Akt phosphorylation is increased during necroptosis, we next examined whether it is still dependent on PI3K and PDK1. Inhibition of PI3K and PDK1 using the specific inhibitors LY249002 and BX912 [36] resulted in the efficient inhibition of cell death and Akt Thr308 phosphorylation (Fig. S7A–D). Likewise, siRNA knockdown of PDK1 protected cells from death and inhibited Akt Thr308 phosphorylation (Fig. S7E,F) Therefore, PI3K and PDK1 activity is still required for non-canonical Akt activation during necroptosis.

### Expression of Constitutively Active Akt, Rescues Necroptosis Under Serum Free Conditions

**Figure 7:**
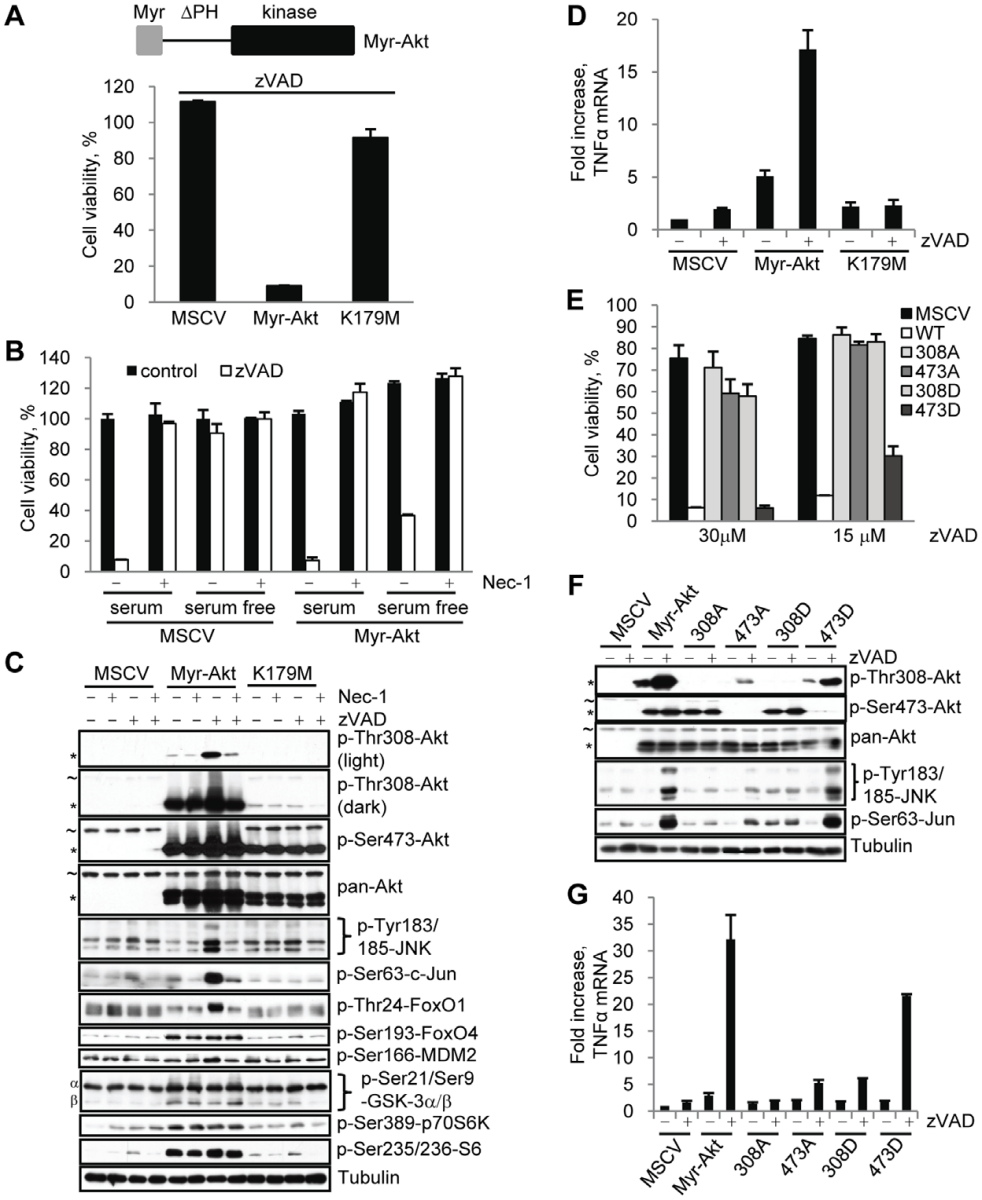
Over expression of constitutively active Akt restores necroptosis under serum free conditions. (A,B) L929 cells were stably infected with empty MSCV retrovirus or viruses encoding Myr-Akt or the catalytically inactive Myr-Akt K179M. Necroptosis was induced by the addition of zVAD.fmk under serum free conditions (A) or serum or serum free conditions with Nec-1 (B). Viability assays were performed after 24 hr. (C) Myr-Akt and Myr-Akt K179M cells were treated with zVAD.fmk and/or Nec-1 under serum free conditions for 9 hr, followed by western blot using the indicated antibodies. Endogenous Akt (∼) and Myr-Akt (*) bands are indicated. (D) L929 cells, stably infected with Myr-Akt and Myr-Akt K179KM, were stimulated with zVAD.fmk for 9 hr under serum free conditions. TNFα mRNA levels were determined by qRT-PCR and normalized using mouse 18S RNA. (E-G) L929 cells expressing Myr-Akt and Ala and Asp mutants of Thr308 and Ser473 were treated with zVAD.fmk under serum free conditions, followed by viability assay at 24 hr (E), western blot at 9 hr (F), or evaluation of TNFα mRNA levels by qRT-PCR at 9 hrs (G). In all graphs, average±SD was plotted.

We used L929 cells stably expressing constitutively active wild type Akt1 (Myr-Akt) or the catalytically inactive mutant K179M in order to further understand the contribution of growth factors and RIP1 kinase to Akt activation during necroptosis. Constitutively active Akt1 (Myr-Akt) was generated as previously described [37] by the addition of a myristoylation signal which provides constitutive localization to the plasma membrane and by the deletion of the auto-inhibitory PH domain (Fig. 7A) resulting in an Akt that is active under serum free. It is important to note that the cells expressing Myr-Akt were viable, grew in a manner indistinguishable from the empty vector control cells, and were not triggered to induce necroptosis by serum starvation (Fig. 7B). This indicates that active Akt alone is not sufficient to induce necroptotic cell death. Under serum free conditions Myr-Akt, but not the K179M mutant, fully restored zVAD.fmk-induced necroptosis (Fig. 7A,B). Nec-1 prevented both Myr-Akt dependent cell death and the necroptosis-specific delayed increase in Akt Thr308 phosphorylation (Fig. 7B,C). Myr-Akt also allowed other zVAD.fmk-dependent events, including activation of JNK and c-Jun phosphorylation (Fig. 7C) and upregulation of TNFα mRNA (Fig. 7D) to occur under serum free conditions, confirming an important role for Akt at the apex of necroptotic signaling. These data demonstrated that the presence of active and membrane localized Akt is sufficient to uncouple Akt activation during necroptosis from growth factor signaling. RIP1 kinase was still able to regulate Akt activation during necroptosis, suggesting that growth factors and RIP1 kinase provide two independent inputs required for Akt changes during necroptosis.

RIP1 kinase-dependent Thr308 phosphorylation of Myr-Akt during necroptosis increased Myr-Akt activity as it did with endogenous Akt (Fig. 5). Phosphorylation of many previously described Akt substrates was increased upon the expression of Myr-Akt, but not the K179M mutant, confirming that these molecules are Akt substrates in L929 cells (Fig. 7C). The effect of zVAD.fmk on their phosphorylation varied, likely due to the increased basal activity of Myr-Akt. Some substrates, including p70S6K, S6, GSK-3 and FoxO4, were fully phosphorylated even in the absence of zVAD.fmk. On the other hand, phosphorylation of FoxO1 and MDM2 was significantly increased in the presence of zVAD.fmk, indicating that necroptotic Thr308 phosphorylation of Myr-Akt still promoted its activity.

Under serum free conditions all zVAD.fmk-induced downstream events (cell death, JNK activation, TNFα production) were dependent on the over expressed Myr-Akt. This allowed us to examine the effects of other Akt mutations on necroptosis. First, we found that membrane localization of Akt is required. Full length Akt or a mutant lacking both the PH domain and the Myr tag did not support the activation of cell death or increased Thr308 phosphorylation following zVAD.fmk addition under serum free conditions (Fig. S8A,B). Second, we found a specific and critical role for Thr308 phosphorylation in the regulation of the necroptotic functions of Akt. It has been reported that Ala mutations at Thr308 and Ser473 cause a reduction in the catalytic activity of Akt, while Asp mutants increase activity [38]. We examined the effect of Ala and Asp mutants at both sites during necroptosis. In our hands, both Asp mutants displayed activity comparable to wild type Akt, while both Ala mutants displayed comparable decreases in activity (Fig. S8C). Despite similar catalytic activities, Thr308 and Ser473 mutants displayed major differences in their ability to promote necroptotic changes (Fig. 7E,F,G). As expected, the S473D mutant, which was phosphorylated on Thr308 after the addition of zVAD, displayed only slightly reduced activity, while S473A was significantly less active in all aspects of necroptosis. S473A was unable to be efficiently phosphorylated on Thr308 possibly due to the inability of the Ala mutated 473 site to be phosphorylated and provide a docking site for PDK1 to phosphorylate Thr308 [39]. Strikingly, both Ala and Asp mutants of Thr308 were significantly less active in promoting cell death, phosphorylation of JNK and c-Jun, and TNFα mRNA. This suggests that T308D, in spite of being an active Akt construct, may not be a perfect mimic of phosphorylation and this mutant form of the kinase may not have sufficient activity to phosphorylate the entire repertoire of substrates in the cells. When tested, T308D did not support the downstream phosphorylation of several substrates that were phosphorylated by the Myr-Akt construct in the presence of zVAD including FoxO1, Foxo4, MDM2, and p70S6K (Fig. S8D). Our model, based on these results, is that necroptosis-specific Thr308 phosphorylation provides a critical link between necroptotic machinery and Akt kinase, allowing Akt to phosphorylate substrates during necroptosis, promote TNFα synthesis, JNK activation and eventual cell death.

### Akt Controls TNFα Production in Other Cell Types

**Figure 8:**
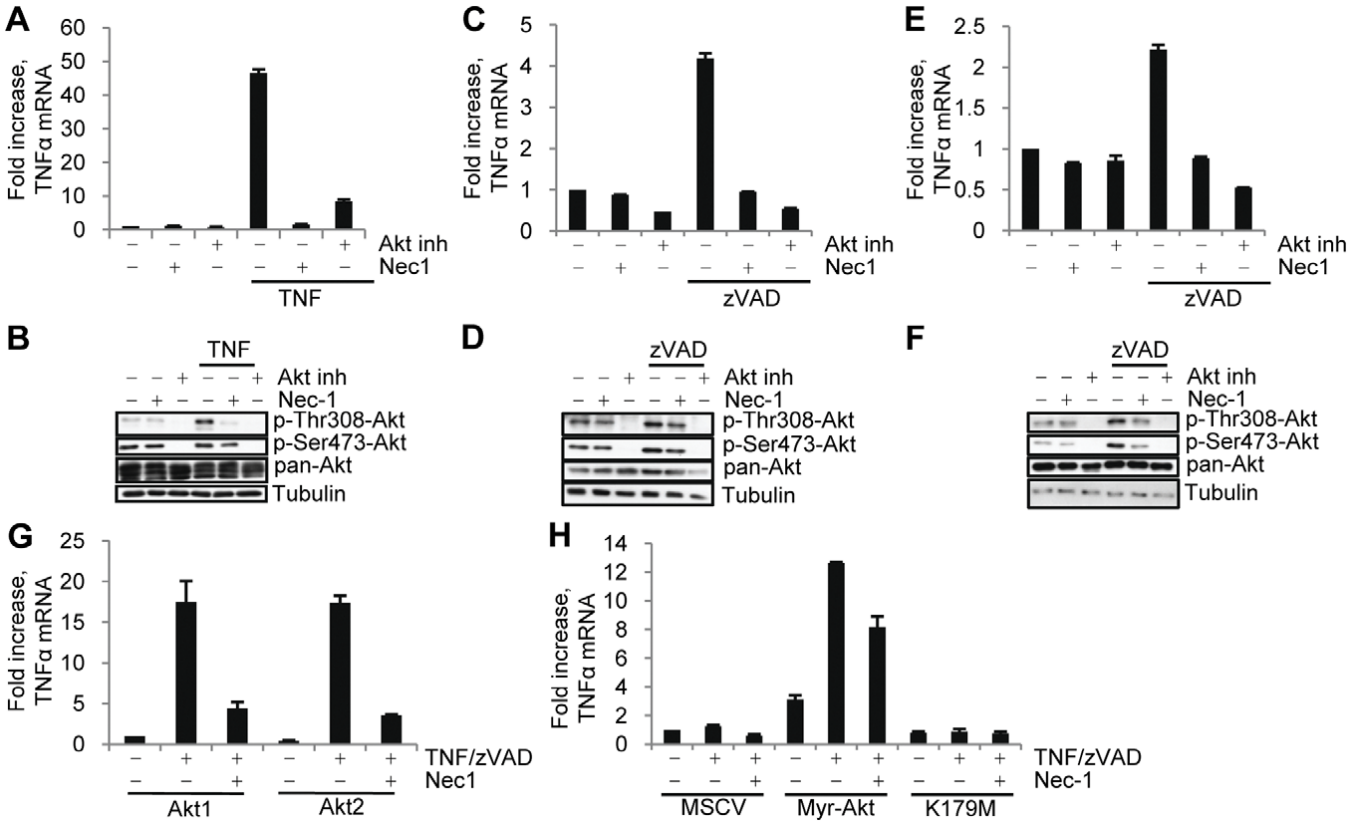
Akt signaling contributes to autocrine TNFα production in multiple cell types. FADD deficient Jurkat cells were treated with TNFα followed by measurement of (A) human TNFα mRNA levels by qRT-PCR and normalized using human 18S RNA or (B) western blot at 9 hr. RAW 264.7 or J774A.1 cells were treated with zVAD.fmk (100 uM or 50 uM respectively) followed by (C,E) measurement of TNFα mRNA levels by qRT-PCR or (D,F) western blot at 9 hr. (G) Akt null mouse lung fibroblasts expressing Myr-Akt or K179M were treated with zVAD.fmk and TNFα followed by measurement of TNFα mRNA levels by qRT-PCR at 9 hr. (H) Mouse lung fibroblasts expressing only endogenous Akt1 or Akt2 were treated with zVAD.fmk and TNFα followed by measurement of TNFα mRNA levels by qRT-PCR at 9 hr.

After establishing the role of RIP1 kinase-dependent signaling to Akt in L929 cells, we sought to expand our study to other cell types that are known to undergo necroptotic cell death. Fas-associated protein with death domain (FADD)-deficient Jurkat T lymphocytes and the macrophage cell lines (J774A.1 and RAW264.7) are other models of necroptosis, which can be induced by stimulation with TNFα or zVAD.fmk, respectively [17]. Similar to L929 cells, a RIP1 kinase dependent increase in the phosphorylation of Thr308 on Akt occurred during necroptosis (Fig. 8B,D,F) in these cell types. Furthermore, TNFα mRNA levels were increased in each of these cell types during necroptosis and efficiently inhibited by both RIP1 and Akt inhibitors (Fig. 8A,C,E). However, inhibition of Akt did not protect these cells from death (Fig. S9A,B,C). These results indicate that regulation of autocrine TNFα synthesis and necroptosis-associated inflammatory signaling may be a more important function of Akt pathway activation by RIP1 kinase in multiple cell types compared to its contribution to cell death.

We next chose to look at the role of Akt in necroptosis in mouse lung fibroblasts. Lung fibroblasts selected to survive after deletion of all three Akt isoforms [40] were resistant to cell death induced by the addition of TNFα and zVAD.fmk. Expression of catalytically active Akt (Myr-Akt) in these cells restored TNFα mRNA production in response to TNFα and zVAD.fmk (Fig. 8G) without re-establishing cell death (Fig. S9D). Consistent with our earlier Akt knockdown data, lung fibroblasts expressing endogenous Akt1 or Akt2 were phosphorylated on Thr308 in response to TNFα and zVAD.fmk (Fig. S9E) and in both cases robust RIP1-dependent TNFα mRNA upregulation occurred under necroptotic conditions (Fig. 8H). These data further support the notion that Akt activity is critical for autocrine TNFα synthesis, even in the absence of necroptotic cell death, indicating an unexpected differentiation between Akt-mediated inflammatory signaling under necroptotic conditions and cell death *per se*.

### Model of RIP1, Akt and JNK Dependent Signaling in Necroptotic L929 Cells

**Figure 9:**
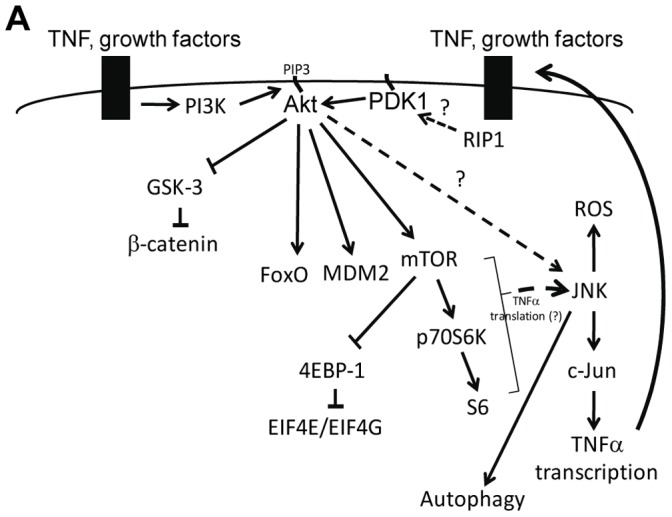
Model of RIP1, Akt and JNK dependent signaling in necroptotic L929 cells. Akt phosphorylation at Thr308 during necroptosis requires inputs from both growth factors and RIP1 kinase. Downstream from Akt, JNK activation leads to TNFα synthesis. Activation of Akt during necroptosis also leads the phosphorylation of several known Akt substrates, such as mTOR, which contribute to the execution of necroptotic death in L929 cells.

In this study we investigated RIP1 kinase-dependent signaling pathways using mouse fibrosarcoma L929 cells that die by necroptosis when treated with the pan-caspase inhibitor zVAD.fmk. Altogether, our results suggest that Akt kinase is specifically engaged in signaling downstream from RIP1 kinase, which leads to a selective increase in its phosphorylation on Thr308, but not Ser473. According to our model (Fig. 9), necroptosis-associated phosphorylation of Akt requires two distinct signals. The first input, which is induced by growth factors, leads to the plasma membrane localization of Akt. Expression of a constitutively membrane-targeted Akt construct, Myr-Akt, overcomes the requirement for growth factors. At the same time, expression of Myr-Akt alone is not sufficient for the induction of necroptosis. A second, RIP1 kinase-dependent input is required for Thr308 phosphorylation of Akt in response to caspase inhibition and is essential for the propagation of the necroptotic signal.

Using Akt inhibitors, knockdown of Akt isoforms, and the expression of Akt mutants, we showed that necroptotic activation of Akt is indispensable for this form of cell death in L929 cells. We also investigated downstream Akt-dependent pathways that contribute to necroptosis. First, we demonstrated that selective necroptotic phosphorylation of Thr308 of Akt is sufficient to increase its activity towards a number of known substrates and Akt effector pathways such as the mTORC1 pathway, which, in turn, contributes to cell death. Second, our data suggested that Akt activation provides a pivotal link connecting RIP1 kinase to known downstream signaling and execution events in necroptotic L929 cells, namely, JNK activation and autocrine TNFα synthesis, a critical event in necroptosis in L929 cells [15].

In order to further test our model, we examined Akt phosphorylation after inhibition of a downstream kinase in the pathway, JNK. However, we found that SP600125, which protected L929 cells from death and inhibited TNFα production (Fig. 2A,B S2A S10A), inhibited both basal and post-treatment phosphorylation levels of Akt at both Ser473 and Thr308 (Fig. S10B). It has been published that SP600125 is a somewhat non-specific inhibitor that may inhibit the p110δ subunit of PI3K [41] and PDK1 [42]. Both of these off-target effects could inhibit basal Akt phosphorylation levels, precluding the use of SP600125 in this system.

Therefore, to examine the role of JNK, we switched to a more specific JNK inhibitor, JNK inhibitor VIII [43], and siRNAs against JNK1 and JNK2 (Fig. S10B–G). As expected, specific inhibition or knockdown of JNK1/2 allowed phosphorylation of Akt on Thr308 while inhibiting the phosphorylation of c-Jun at Ser63 (Fig. S10B,E), agreeing with our model. It did not, however, lead to a reduction in TNFα production or cell death (Fig. S10C,D,F,G), suggesting that earlier data with SP600125 protection (Fig. 2) could reflect off-target effects of this molecule, rather than JNK inhibition. Previous reports also suggested a critical role for c-Jun in necroptosis and autocrine TNFα synthesis [13], [14], [15] and we confirmed these conclusions using c-Jun siRNA knockdown (Fig. S10H–J). Notably, in this case, Thr308 phosphorylation was reduced after the induction of necroptosis. Thus, autocrine TNFα production, dependent on c-Jun, may create a feedback loop that contributes to the delayed activation of Akt. It is also important to note that we observed an overall increase in the protein level of c-Jun after treatment of L929 cells with zVAD.fmk or TNFα, which was both Akt and mTOR-dependent (Fig. 6E,F). These new data led us to an unexpected, but important conclusion that c-Jun is critical for necroptosis, while JNK activity may serve as a useful marker of pathway activation, but may be either redundant (e.g. phosphorylation of c-Jun on a site other than Ser63 may occur [44]) or dispensable functionally. In addition, researchers need to use caution when using SP600125 due to potantial off-target effects.

## Discussion

Altogether, our results suggest that Akt kinase is specifically engaged in the signaling downstream from RIP1 kinase, which exerts its activity through promoting a selective increase in Akt phosphorylation on Thr308. This provides a link connecting RIP1 kinase to downstream signaling and execution events during necroptosis in L929 cells, including JNK activation, autocrine TNFα synthesis and eventual cell death. According to our model, phosphorylation of Akt requires two distinct signals. The first input, which is induced by growth factors, leads to the plasma membrane localization of Akt. Expression of constitutively active membrane-targeted Myr-Akt overcomes this requirement. At the same time, expression of Myr-Akt is not sufficient for the induction of necroptosis or efficient activation of JNK and TNFα synthesis. A second, RIP1 kinase-dependent input is required for Thr308 phosphorylation of Akt, which in turn is required for necroptotic signaling. Necroptotic phosphorylation of Thr308 of Akt is sufficient to increase its activity towards a number of known substrates in L929 cells and our data reveal that the Akt effector pathway downstream of mTORC1 contributes to necroptosis, thereby identifying a new mediator of this form of cell death.

Our results raise some important mechanistic questions relevant to the specific regulation of Akt during necroptosis. First, what is the mechanism of the RIP1-dependent increase in Akt Thr308 phosphorylation? One possibility is that RIP1 kinase inhibits a phosphatase that targets Thr308. To our knowledge, PP2A is the only enzyme established to specifically dephosphorylate this residue [45]. However, we did not observe any effect of the PP2A inhibitor, okadaic acid, on Thr308 phosphorylation or activation of necroptosis in L929 cells. Another possibility is that the increase in Thr308 results from RIP1 kinase targeting PDK1, Akt or scaffolding factors that bring these two kinases together. Interestingly, we observed phosphorylation of Akt by recombinant RIP1 kinase *in vitro* on Thr146, 195/197, and 435 and Ser381 residues. Furthermore, mutating these residues to Ala in Myr-Akt leads to the loss of its ability to promote necroptosis. However, we were not able to confirm phosphorylation of these residues on endogenous Akt in L929 cells using either mass spectrometry or western blotting with a phospho-specific antibody raised against Thr435 peptide, suggesting that direct phosphorylation of Akt by RIP1 likely represents an *in vitro* artifact and does not reflect endogenous regulation. Second, what are the key substrates of Akt that promote necrotic death and TNFα synthesis? On the one hand, our data suggest new roles for Akt effector pathways mediated by mTORC1 in necroptotic control. On the other hand, we have observed only modest changes in mTORC1 activity under necroptotic conditions, suggesting that additional Akt substrates are likely to be involved. This warrants a re-evaluation of the roles of additional Akt substrates in necroptotic death, since no such connections have been established. Similarly, the mechanisms connecting mTORC1 to JNK remain to be elucidated. While there are some recent examples of mTORC1-dependent regulation of JNK, e.g. following ER stress [46], the exact mechanisms during necroptosis remain to be established. Given the activation of JNK by TNFα and the importance of mTORC1-dependent translational control in necroptosis, one possibility is that mTORC1 contributes to the translation of TNFα and forms a positive feed forward loop with JNK.

Akt’s role as a key inhibitor of apoptosis is well documented, however, evidence of its contribution as a mediator of cell death under various circumstances has begun to emerge as well [45], [47]. Our data demonstrates a new mode of necrosis-specific regulation of Akt by RIP1 kinase. Importantly, while it is possible that necroptosis-specific targets of Akt exist, this regulation clearly involves a number of well established Akt targets including mTORC1, and potentially, GSK-3, FoxO1/4, and MDM2. Therefore, it may no longer be safe to assume that activation of Akt universally reflects pro-survival signaling nor that its inhibition will lead to more cell death. It is tempting to speculate that rather than serving a universally pro-survival role, the Akt pathway may function to promote cell fates alternative to apoptosis, ranging from survival to non-apoptotic cell death. The final decision between survival and death may depend on additional, Akt-independent inputs, such as the status of RIP1 kinase, expression of particular oncogenic factors [45] or excessive metabolic stress [47].

Another mechanism that should be considered in conjunction with the regulation of cell death by Akt is autophagy. Akt activation leads to the inhibition of autophagy through activation of mTOR [48]. The role of autophagy in cell death in general is very complex and it can both promote and inhibit necroptosis in various situations. Several studies suggested that activation of autophagy promotes necroptosis induced by zVAD.fmk in L929 cells [11], [14]. Others, including ourselves in unpublished data, have found that inihibition of autophagy promotes necroptosis by TNFα [49]. This suggests that the inhibition of autophagy by Akt or mTOR in our system may contribute to necroptosis induced by TNFα, however, it is more difficult to reconcile with the positive role of these proteins in zVAD-induced death. Clearly, further identification of the factors differentiating between pro-death and pro-survival autophagy in mammalian cells is required to better understand its role in the regulation necroptosis by Akt pathway.

Importantly, our data revealed that RIP1 kinase signaling to Akt is a general feature of necroptotic signaling that is observed in multiple cell types. At the same time, the significance of this connection varies in a cell type specific fashion. Importantly, in mouse lung fibroblasts, FADD-deficient Jurkat cells, and macrophages, Akt signaling contributed more prominently to an increase in TNFα synthesis, rather than cell death *per se*, unlike its role in L929 cells. A recent study [15] has demonstrated that, in addition to its role in necroptosis, RIP1 plays an important role in mediating the production of TNFα. These data emphasize the emerging complexity of necroptotic signaling mechanisms and highlight the major contribution of Akt to increased inflammatory signaling, specifically accompanying this form of regulated necrosis [5], [6], [10].

Robust inflammation is one of the most important consequences of necrotic cell death as well as its regulated subtype, necroptosis, both *in vitro* and *in vivo* [1], [5], [6], [50], [51]. Our results highlight an important notion that inflammation not only passively accompanies necroptosis in a variety of cellular systems by the virtue of rapid loss of plasma membrane integrity characteristic for necrotic cell death, but also that it is an intrinsic and regulated component of necroptosis due to the specific activation of TNFα synthesis by RIP1/Akt kinases. Therefore, this pathway may represent a new molecular target for the inhibition of pathologic inflammatory signaling. Initial *in vivo* data appears to support this notion. Two recent papers showed that the loss of control over RIP1/RIP3 kinase activities by FADD and caspase-8 in epithelial cells unleashes a feed forward cycle of necroptosis and TNFα production, resulting in the development of intestinal inflammation in mice and, possibly, in patients with Crohn’s disease [4], [5]. This increased production of TNFα during necroptosis may also be important for acute necrotizing diseases, such as necrotizing pancreatitis and acute bacterial infections, where hyper-acute inflammation accompanying necrotic cell death is the primary cause of multiple organ failure and patient death. Along these lines, another recent paper by Duprez et al. has shown that RIP1 and RIP3 mediate the cellular damage introduced by TNF-induced SIRS [6]. The role of RIP1 kinase in acute and chronic inflammatory diseases warrants further investigation, as efficient and specific RIP1 kinase inhibitors may offer therapeutic benefit for treating these conditions.

## Materials and Methods

### Reagents and Chemicals

Necrostatin analogs were synthesized as previously described [23], [24]. The following reagents and final concentrations (unless otherwise specified in the text/figures) were used in the experiments: Akt inhibitor VIII (10 µM, Calbiochem), MK-2206 (10 µM, Selleck Chem), Triciribine (100 µM, National Cancer Institute), SP600125 (10 µM, Calbiochem), JNK inhibitor VIII (10 µM, Calbiochem), UO126 (10 mM, Cayman Chem), PD169316 (10 µM, Calbiochem), LiCl (10 mM, Sigma), SB216763 (10 µM, Calbiochem), BX912 (10 µM, Axon Med Chem), PF-4706871 (Sigma), rapamycin (100 nM, Santa Cruz), PI-103 (10 µM, Calbiochem), Torin-1 (500 nM, gift of Dr. Nathanael Grey (Harvard Medical School), LY249002 (10 µM, Cell Signaling), PD173074 (2 µM, Cayman Chem), PD166866 (20 µM, Calbiochem), 4EGI-1 (50 µM, Calbiochem). Pan-caspase inhibitor zVAD.fmk (20–30 µM) was purchased from Bachem. Human and mouse TNFα (10 ng/ml), human bFGF (25 ng/ml), EGF (50 ng/ml), PDGF-BB (20 ng/ml), and IGF-1 (50 ng/ml) were from Cell Sciences or Peprotech. All other reagents were from Sigma.

### DNA

Cloning of Myr-Akt1, containing c-terminal FLAG tag, has been described [52]. Myr-Akt1-FLAG was amplified by PCR and subcloned into the BglII and EcoRI sites of pMSCV-puro retroviral vector (Invitrogen). Mutant versions of Myr-Akt1 were generated using the same strategy.

### Antibodies

The following antibodies were used: phospho-Akt (Thr308) (clone C31E5E) rabbit mAb, phospho-Akt (Ser473) (clone D9E) XP rabbit mAb, Akt (pan) (clone C67E7) rabbit mAb, Akt1 (clone C73H10) rabbit mAb, Akt2 (clone D6G4) rabbit mAb, Akt3 (clone 62A8) rabbit mAb, phospho-JNK (Thr183/Tyr185) (81E11) rabbit mAb, SAPK/JNK rabbit pAb, phospho-c-Jun (Ser63) II rabbit pAb, c-Jun (60A8) rabbit mAb, α-tubulin (clone DM1A) mouse mAb, phospho-FoxO1 (Thr24)/FoxO3a (Thr32) rabbit pAb, FoxO1 (L27) rabbit pAb, phospho-FoxO4 (Ser193) rabbit pAb, FoxO4 rabbit pAb, phospho-MDM2 (Ser166) rabbit pAb, phospho-GSK-3α/β (Ser21/9) rabbit pAb, phospho-p70 S6 Kinase (Thr389) (clone 108D2) rabbit mAb, phospho-S6 Ribosomal Protein (Ser235/236) (clone D57.2.2E) XP rabbit mAb, S6 Ribosomal Protein (clone 54D2) mouse mAb, phospho-4E-BP1 (Thr37/46) rabbit pAb, mTOR (clone 7C10) rabbit mAb, PDK1 rabbit pAb (all Cell Signaling), MDM2 rabbit pAb (Bioworlde).

### QPCR Primers

Mouse TNFα: forward 5′-CCCTCACACTCAGATCATCTTCT-3′, reverse 5′-GCTACGACGTGGGCTACAG-3′;mouse 18S: forward 5-′ ATAACAGGTCTGTGATGCCCTTAG-3, reverse 5′-CTAAACCATCCAATCGGTAGTAGC-3′;human TNFα: forward 5′- ATGAGCACTGAAAGCATGATCC-3′, human TNFα: reverse 5′-GAGGGCTGATTAGAGAGAGGTC-3′; human 18S: forward 5′- CAGCCACCCGAGATTGAGCA -3, human 18S: reverse 5′-TAGTAGCGACGGGCGGTGTG-3′.

### Cell Lines

L929 and FADD-deficient Jurkat cells were obtained from ATCC. Lung fibroblasts were a generous gift of Dr. Philip Tsichlis (Tufts University) [53]. J774A.1 (ATCC) cells and RAW264.7 (ATCC) cells were generous gifts of Junying Yuan (Harvard University) and Alexander Poltorak (Tufts University), respectively. Cells were maintained in DMEM supplemented with 10% fetal bovine serum (FBS) and 1% antibiotic-antimycotic mixture (Invitrogen). The mouse lung fibroblast media was additionally supplemented with L-glutamine, non-essential amino acids, and sodium pyruvate. Jurkat cells were maintained in RPMI1640, supplemented with 10% FetalPlex (Gemini) and 1% antibiotic-antimycotic.

### Cell Viability Experiments

Cells were seeded into white clear bottom 96 well plates at the density of 1×10^4^ cells/well and treated as described for western blot experiments. Cell viability was determined using CellTiter-Glo Cell Viability Assay (Promega). Experiments were performed in duplicate or triplicate. Viability of the control untreated cells was set as 100%. Relative viability of cells, induced to undergo necroptosis and treated with the compound relative to the control compound-treated cells, was determined and plotted to exclude the possible effects of non-specific toxicity of the small molecules.

### siRNA Knockdown

siRNAs were purchased from Dharmacon. Mouse ribosomal S6 protein (L-040893-00 and L-045791-00), mouse Akt1 (L-040709-00), mouse Akt2 (L-040782-00), mouse Akt3 (L-040891-00), mouse mTOR (L-065427-00), mouse PDK1 (L-040658-00), non-coding control (D-001810-10-05), mouse Mapk8 (J-040128-05), mouse Mapk9 (J-040134-05), mouse Jun (L-043776-00). siRNA were transfected using RNAiMAX reagent (Invitrogen), according to manufacturer’s recommendations. After 72 hr, cells were treated with zVAD.fmk or TNFα for 9 hr (RNA or Western blot) or 24 hr (cell viability).

### Western Blot

For Western blot, 4×10^5^ adherent cells (1×10^6^ Jurkat cells) were seeded into 35 mm^2^ dishes. After 24–48 hr, cells were stimulated with 30 µM zVAD.fmk or 10 ng/ml mouse TNFα. For treatments under serum free conditions, cells were serum starved for 24 hr prior to the addition of growth factors, 20 µM zVAD.fmk or 10 ng/ml mouse TNFα. Cells were harvested in 1×RIPA buffer (Cell Signaling) supplemented with 50 µg/ml phenylmethanesulfonylfluoride. After brief sonication, cell lysates were spun down for 15 min at 14,000×rpm. Protein concentrations were measured using the Pierce 660 nm Assay Reagent (Pierce). Equal amounts of proteins were boiled for 5 min at 95°C. Western blotting was performed according to standard protocols. Briefly, SDS-PAGE gels were transferred to PVDF membrane, blocked in 3% milk or 5% bovine serum albumin (BSA) in TBST buffer for 30 min at room temperature. Primary antibodies were incubated in 5%BSA/TBST overnight at 4°C. Secondary antibodies were incubated in TBST for 30 min at room temperature. Luminata (Millipore) ECL reagents were used to develop the signals. In some cases, membranes were stripped using OneMinute stripping buffer (GM Biosciences) and reprobed with new antibodies.

### qRT-PCR

Cells were treated as described for Western blots. Total RNA was isolated using ZR Miniprep kit (Zymo Research). 1 µg of RNA was converted to cDNA using random primers (M-MuLV cDNA kit, New England Biolabs). 1 µL of cDNA was used with 500 pM primers in qPCR reactions. Reactions were performs using SYBRGreen 2×Master mix (SABiosciences) in a LightCycler480 (Roche).

### Stable Infection of Myr-Akt1

To generate MSCV retroviruses, HEK293FT cells (Invitrogen) were transfected with 2 µg of viral DNA and 1 µg of gal/pol and VSV-G accessory plasmids in 6 well plates using GenJet transfection reagent (Signagen Labs). Virus-containing media was collected 72 hr later, filtered through 0.45 µm filter and applied to L929 cells with 8 µg/ml polybrene. Cells were selected and maintained in 10 µg/ml puromycin.

### ELISAOne Assay

ELISAOne assays (TGRBio, Hindmarsh, Australia) were performed according to manufacturer’s protocol with the following modifications. Cell lysates were prepared in RIPA buffer as described for Western blots. Five microliters of samples were diluted in 45 µL of ELISAOne lysis buffer prior to analysis. Primary antibodies to phopsho-Thr308 and phopsho-Ser473 were incubated with the samples for 2 hr at room temperature. Primary antibody to pan-Akt was incubated overnight at 4°C. Signals for phospho-antibodies were normalized based on pan-Akt values.

### TNFα ELISA

Mouse TNFα Quantikine ELISA assays (R&D Systems) were performed according to manufacturer’s descriptions. Cell lysates were prepared from 3×10^6^ cells plated and treated in a 10 cm^2^ dish.

### In vitro Akt Kinase Assay

Akt kinase activity was measured using the Akt kinase assay kit (nonradioactive) from Cell Signaling Technology. In brief, Myr-Akt was immunoprecipitated from L929 cells using anti-FLAG M2 magnetic beads (Sigma). The *in vitro* assay was performed in the presence of a GSK fusion protein substrate. Phosphorylation of the GSK fusion protein was visualized by western blot.

## Supporting Information

Figure S1 **Necroptosis, induced by zVAD.fmk or TNFα, was inhibited by Nec-1 but not Nec-1i.** (A) Structures of 7-Cl-O-Nec-1 (Nec-1) and the inactive analog, 7-Cl-O-Nec-1i (Nec-1i). (B, D) L929 cells were treated with zVAD.fmk or TNFα in the presence of Nec-1 or Nec-1i. (C, E) L929 cells were serum starved followed by treatment with bFGF/zVAD.fmk in the prescence of either Nec-1 or Nec-1i. (B, C) Cell viability was measured 24 hrs post-treatment. (D, E) Cells were harvested for western blot 9 hrs post-treatment. In all graphs, average±SD was plotted.
(TIF)

Figure S2 **Akt contributes to death by necroptosis.** (A) L929 cells treated with zVAD.fmk or TNFα in the presence of the JNK inhibitor, SP600125. Cells were analyzed for cell viability 24 hrs post-treatment. (B) L929 cells were treated with zVAD.fmk or TNFα or bFGF/zVAD.fmk (serum free conditions) in the presence of the Akt inhibitors (Akt inhibitor VIII 10 µM, MK2206 10 µM or Triceribine (TCN) 100 µM) and cell viability was measured 24 hrs post-treatment. (C) Mouse lung fibroblasts expressing either Akt1, Akt2, or Akt3 and L929 lysates were harvested and western blotted using the indicated antibodies. In all graphs, average±SD was plotted.
(TIF)

Figure S3 **RIP1 kinase-dependent increase in Akt Thr308 phosphorylation during necroptosis.** (A, B) L929 cells treated with zVAD.fmk (A) or TNFα (B) for the indicated period of time followed by assessment of cell viability. (C) Cells were serum starved followed by treatment with IGF alone or IGF/zVAD.fmk and samples were collected at the indicated time points for western blot. (D,E) L929 cells were stimulated with bFGF and/or zVAD.fmk and Nec-1 (N1) for the indicated periods of time. Samples were analyzed using phospho-Thr308, phospho-Ser473 and pan-Akt ELISAOne assays. Phospho-signals were normalized to pan-Akt. Fold induction over control cells is plotted. In all graphs, average±SD was plotted.
(TIF)

Figure S4 **Growth factor independent activation of Akt Thr308 phosphorylation by TNFα.** (A) Necroptosis was induced by zVAD.fmk or TNFα in the presence of 2 µM PD173074 or 20 µM PD166866 for 9 hrs followed by western blot. (B,C) Cells were stimulated with TNFα under normal serum (B) or serum free (C) conditions for the indicated periods of time followed by western blot.
(TIF)

Figure S5 **Downstream Akt signaling contributes to the control of necroptosis.** (A,B) L929 were stimulated with zVAD.fmk (A) TNFα (B) for the indicated periods of time, followed by western blot using indicated antibodies. (C,D) L929 were stimulated with TNFα or zVAD.fmk in the presence of the indicated concentrations of PF-4706871. Viability was determined after 24 hr (C). Western blot samples were collected after 9 hr (D). (E) L929 cells were transfected with S6 siRNAs. After 48 hr, necroptosis was induced by TNFα and zVAD.fmk for 24 hr. Inset, levels of S6 were determined 48 hr after transfection. In all graphs, average±SD was plotted.
(TIF)

Figure S6 **Akt and mTORC1 contribute to autocrine TNFα synthesis and JNK activation during necroptosis.** (A,B) L929 cells were stimulated by zVAD.fmk (A) and human TNFα (B) for 9 hr. Cell lysates were subjected to mouse TNFα ELISA. (C) L929 cells were stimulated by zVAD.fmk and TNFα in the presence of either Nec-1 followed by measurement of TNFα mRNA levels by qRT-PCR at 9 hr. (D) L929 cells were stimulated by zVAD.fmk and TNFα in the presence of Akt inh VIII (10 µM), MK2206 (10 µM) and TCN (100 µM) followed by western blot at 9 hrs. (E) Cells were stimulated with TNFα for 15 min or 9 hr in the presence of Nec-1, Akt inh VIII and rapamycin. Western blot samples were collected after 9 hr. (F) L929 cells were stimulated with bFGF and zVAD.fmk (serum free conditions) for 15 min and 9 hr in the presence of Akt inh. VIII and analyzed by western blot. (G) L929 cells were stimulated by zVAD.fmk in the presence of Nec1, Akt inh VIII, or rapamycin followed by western blot at 9 hrs. In all graphs, average±SD was plotted.
(TIF)

Figure S7 **PI3-Kinase and PDK1 mediate the increase in Akt Thr308 phosphorylation under necroptotic conditions.** (A-D) L929 cells were stimulated by zVAD.fmk and TNFα in the presence of LY249002 (A,B) or BX912 (C,D). Viability assays were performed after 24 hr (A,C). Western blot samples were collected after 9 hr. (B,D). (E,F) L929 cells were transfected with PDK1 siRNAs. After 72 hr, necroptosis was induced by TNFα or zVAD.fmk. Viability assays were performed after 24 hr (E). Western blot samples were collected after 9 hr (F). In all graphs, average±SD was plotted.
(TIF)

Figure S8 **Constitutively active Myr-Akt promotes necroptosis under serum free conditions.** (A,B) L929 cells expressing Myr-Akt, full length Akt and a mutant lacking the PH domain were treated with zVAD.fmk under serum free conditions, followed by viability measurement at 24 hr (A) or western blot at 9 hr (B). * - non-specific band, coinciding with the migration of Myr-Akt was detected by some lots of the p308 antibody. (C) L929 cells expressing Myr-Akt and Ala and Asp mutants of Thr308 and Ser473 were immunoprecipitated from L929 cells and their in vitro catalytic activity towards GSK-3β peptide was determined. (D) L929 cells expressing Myr-Akt or the T308D mutant were treated with zVAD.fmk for 9 hrs under serum free conditions followed by western blot analysis. In all graphs, average±SD was plotted.
(TIF)

Figure S9 **Akt signaling contributes to autocrine TNFα production in multiple cell types.** (A) FADD-deficient Jurkat cells were treated with TNFα in the presence of Nec-1 or Akt inh VIII. Cell viability was assayed after 24 hrs. (B,C) RAW 264.7 (B) or J774A.1 (C) were treated with zVAD.fmk (100 uM or 50 uM respectively). Cell viability was assayed after 24 hrs. (D,E) Akt deficient mouse lung fibroblasts stably expressing Myr-Akt or Myr-Akt K179M mutant, were stimulated with TNFα and zVAD.fmk under serum free conditions for 24 hr, followed by cell viability assay or (D) western blot analysis (E). (F) Mouse lung fibroblasts expressing one isoform of Akt (Akt1 or Akt2) were treated with zVAD.fmk and TNFα followed by cell viability assay. In all graphs, average±SD was plotted.
(TIF)

Figure S10 **JNK and c-Jun differentially contribute to autocrine TNFα production and cell death.** (A) Cells were treated with TNFα or zVAD.fmk with or without SP600125 (SP6) followed by evaluation of TNFα mRNA levels by qRT-PCR at 9 hrs. (B) L929 cells were treated with TNFα for 9 hrs in the presence of the JNK inhibitor SP600125 (SP6), Nec-1, JNK inh. VIII, or Akt inh. VIII and analysed by western blot. (C) Cells were treated with TNFα with or without JNK inhibitor VIII followed by evaluation of TNFα mRNA levels by qRT-PCR at 9 hrs. (D) Cells were treated with zVAD.fmk or TNFα with either SP600125 or JNK inh VIII followed by viability assay at 24 hr. (E-G) L929 cells transfected with JNK1 and JNK2 siRNAs for 72 hrs were treated with zVAD.fmk or TNFα followed by western blot at 9 hr (E), evaluation of TNFα mRNA levels by qRT-PCR at 9 hrs (F), or viability assay at 24 hr (G), (H-J) L929 cells transfected with c-jun siRNAs for 72 hrs were treated with zVAD.fmk or TNFα followed by western blot at 9 hr (H), evaluation of TNFα mRNA levels by qRT-PCR at 9 hrs (I), or viability assay at 24 hr (J), In all graphs, average±SD was plotted.
(TIF)
