# Supplementary figures and images for: Akt Regulates TNFα Synthesis Downstream of RIP1 Kinase Activation during Necroptosis
Source: PLoS One. 2013 Mar 1;8(3):e56576. doi: 10.1371/journal.pone.0056576 (PMC3585731; doi:10.1371/journal.pone.0056576)

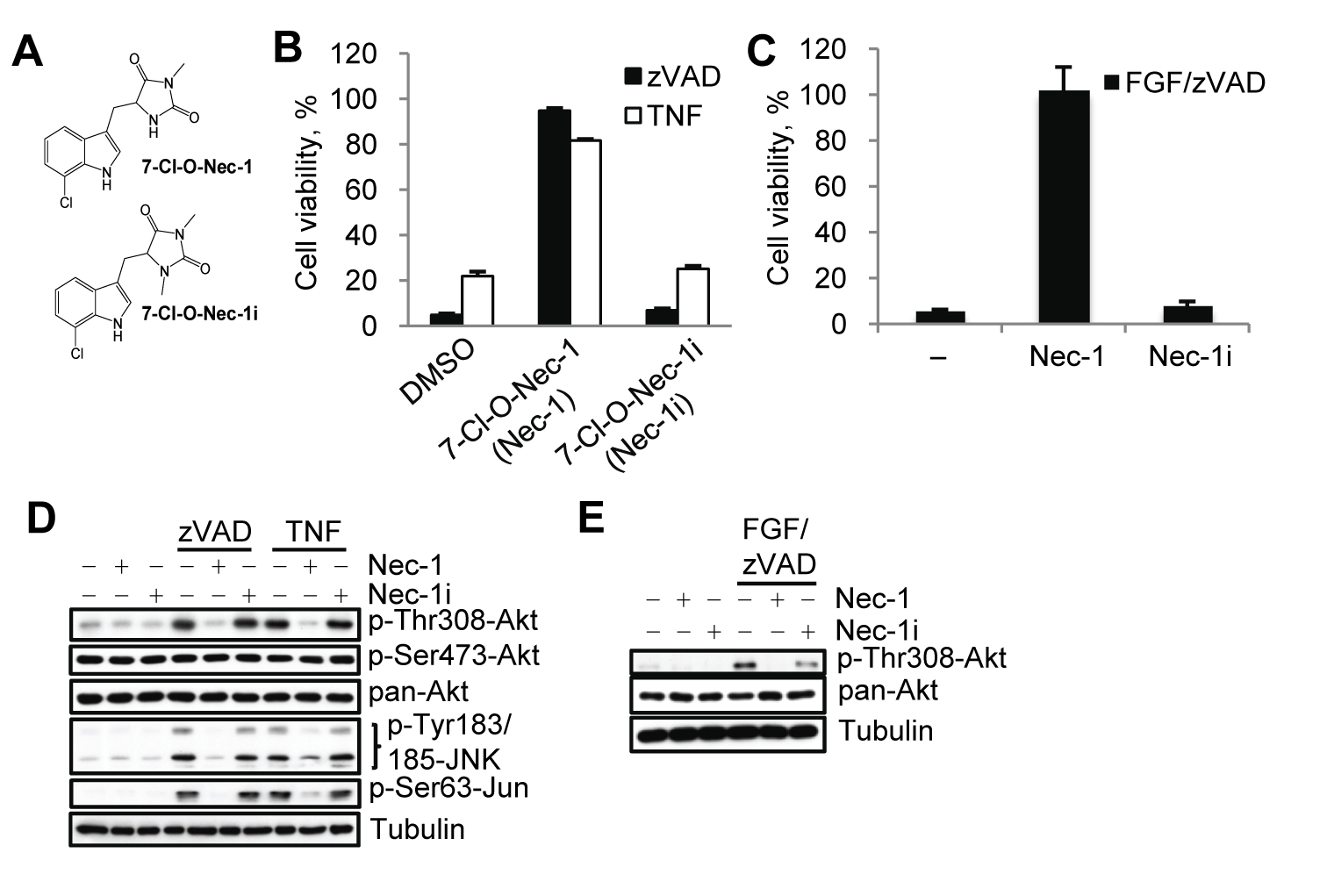

Supplement: Figure S1 — Necroptosis, induced by zVAD.fmk or TNFα, was inhibited by Nec-1 but not Nec-1i. (A) Structures of 7-Cl-O-Nec-1 (Nec-1) and the inactive analog, 7-Cl-O-Nec-1i (Nec-1i). (B, D) L929 cells were treated with zVAD.fmk or TNFα in the presence of Nec-1 or Nec-1i. (C, E) L929 cells were serum starved followed by treatment with bFGF/zVAD.fmk in the prescence of either Nec-1 or Nec-1i. (B, C) Cell viability was measured 24 hrs post-treatment. (D, E) Cells were harvested for western blot 9 hrs post-treatment. In all graphs, average±SD was plotted. (TIF) [file pone.0056576.s001.tif]

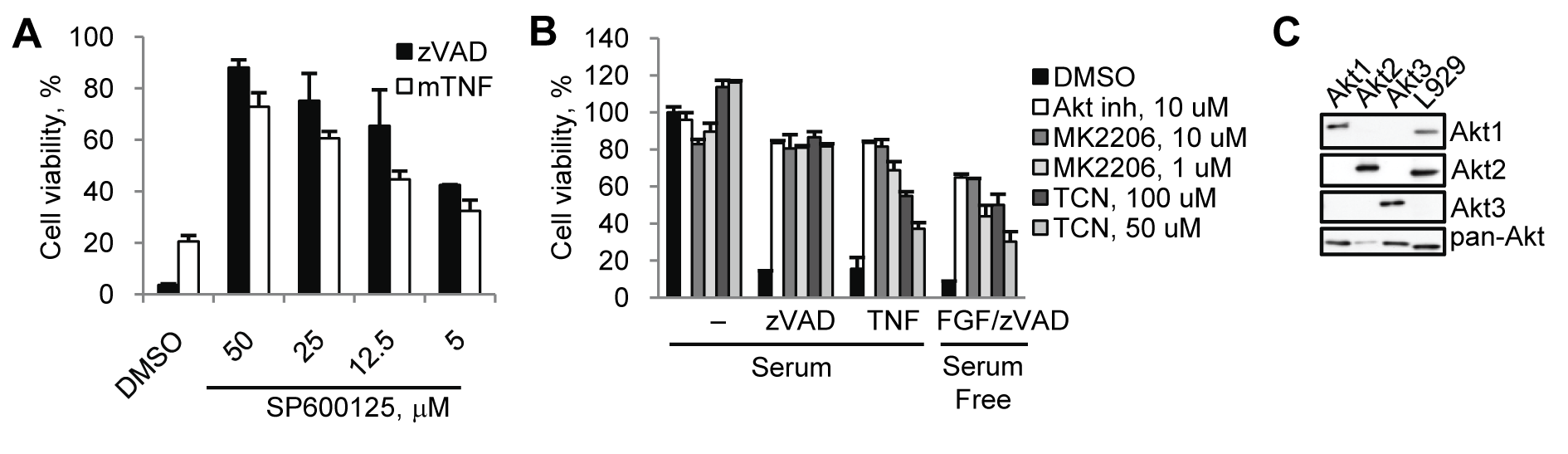

Supplement: Figure S2 — Akt contributes to death by necroptosis. (A) L929 cells treated with zVAD.fmk or TNFα in the presence of the JNK inhibitor, SP600125. Cells were analyzed for cell viability 24 hrs post-treatment. (B) L929 cells were treated with zVAD.fmk or TNFα or bFGF/zVAD.fmk (serum free conditions) in the presence of the Akt inhibitors (Akt inhibitor VIII 10 µM, MK2206 10 µM or Triceribine (TCN) 100 µM) and cell viability was measured 24 hrs post-treatment. (C) Mouse lung fibroblasts expressing either Akt1, Akt2, or Akt3 and L929 lysates were harvested and western blotted using the indicated antibodies. In all graphs, average±SD was plotted. (TIF) [file pone.0056576.s002.tif]

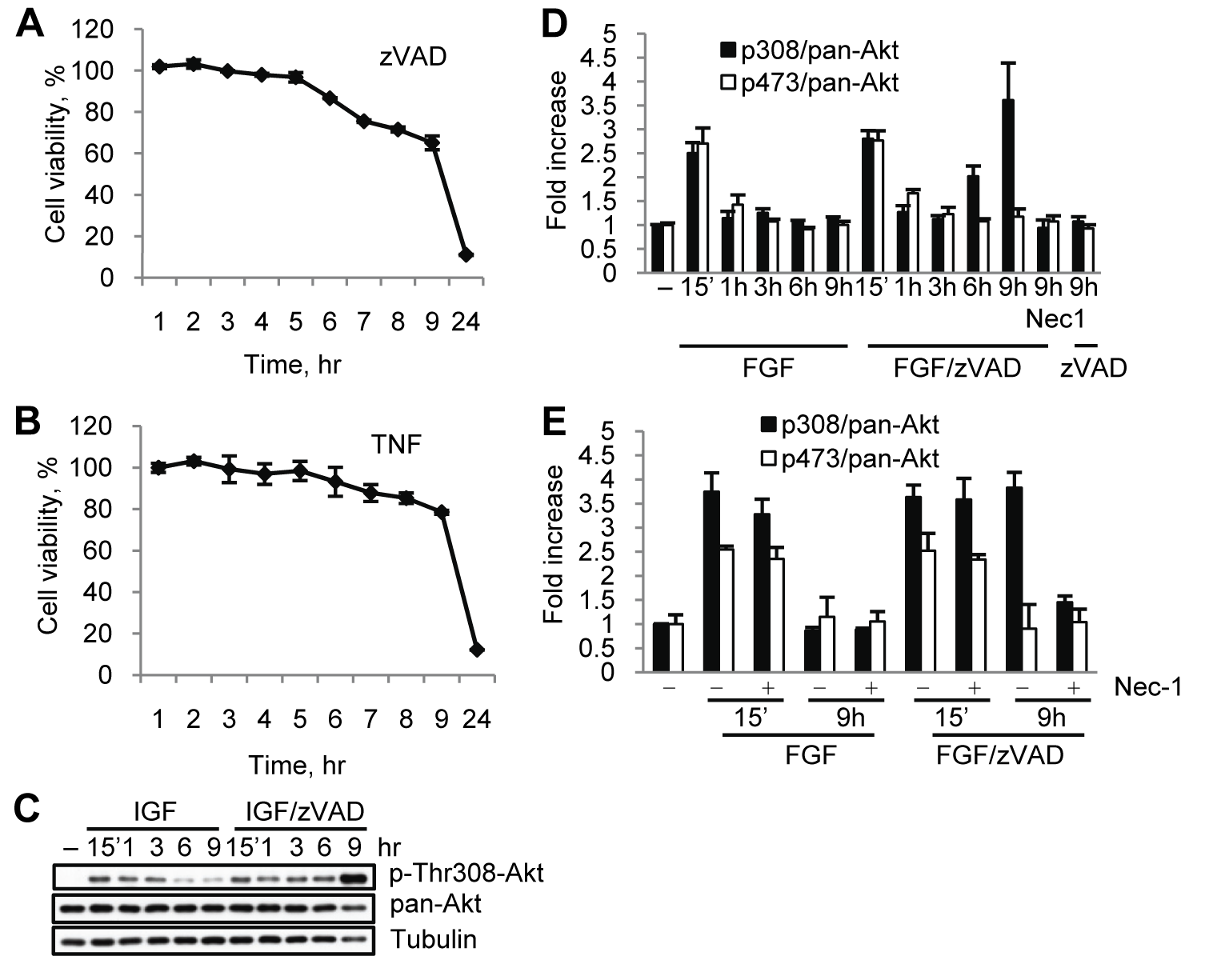

Supplement: Figure S3 — RIP1 kinase-dependent increase in Akt Thr308 phosphorylation during necroptosis. (A, B) L929 cells treated with zVAD.fmk (A) or TNFα (B) for the indicated period of time followed by assessment of cell viability. (C) Cells were serum starved followed by treatment with IGF alone or IGF/zVAD.fmk and samples were collected at the indicated time points for western blot. (D,E) L929 cells were stimulated with bFGF and/or zVAD.fmk and Nec-1 (N1) for the indicated periods of time. Samples were analyzed using phospho-Thr308, phospho-Ser473 and pan-Akt ELISAOne assays. Phospho-signals were normalized to pan-Akt. Fold induction over control cells is plotted. In all graphs, average±SD was plotted. (TIF) [file pone.0056576.s003.tif]

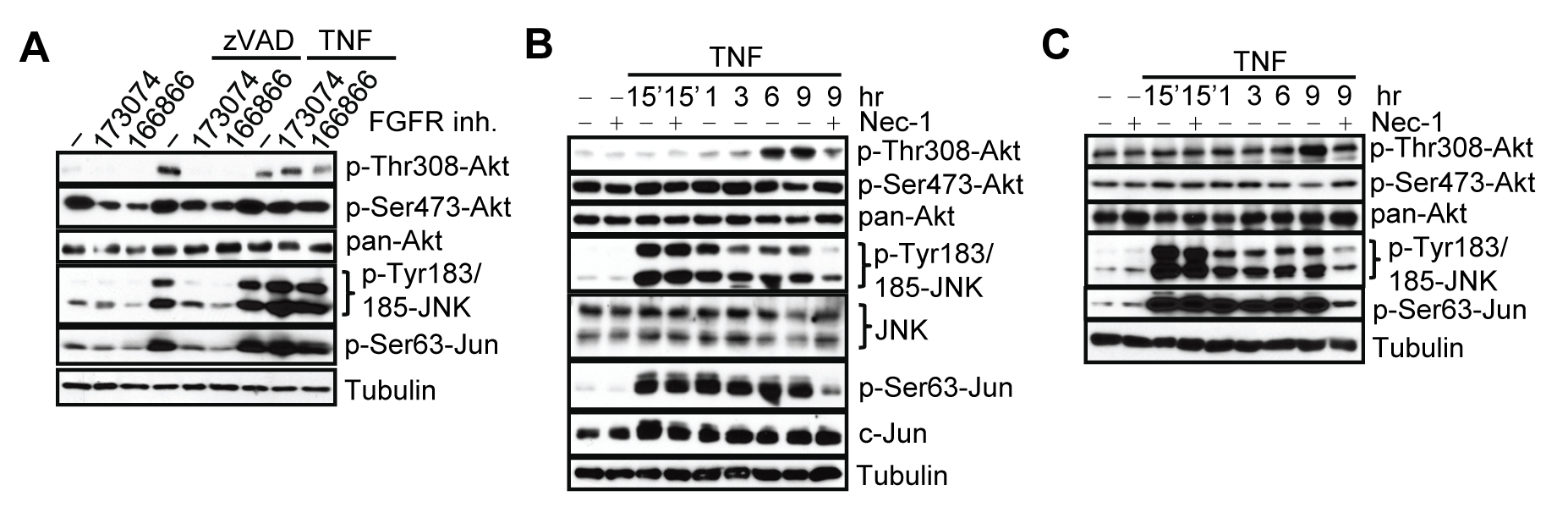

Supplement: Figure S4 — Growth factor independent activation of Akt Thr308 phosphorylation by TNFα. (A) Necroptosis was induced by zVAD.fmk or TNFα in the presence of 2 µM PD173074 or 20 µM PD166866 for 9 hrs followed by western blot. (B,C) Cells were stimulated with TNFα under normal serum (B) or serum free (C) conditions for the indicated periods of time followed by western blot. (TIF) [file pone.0056576.s004.tif]

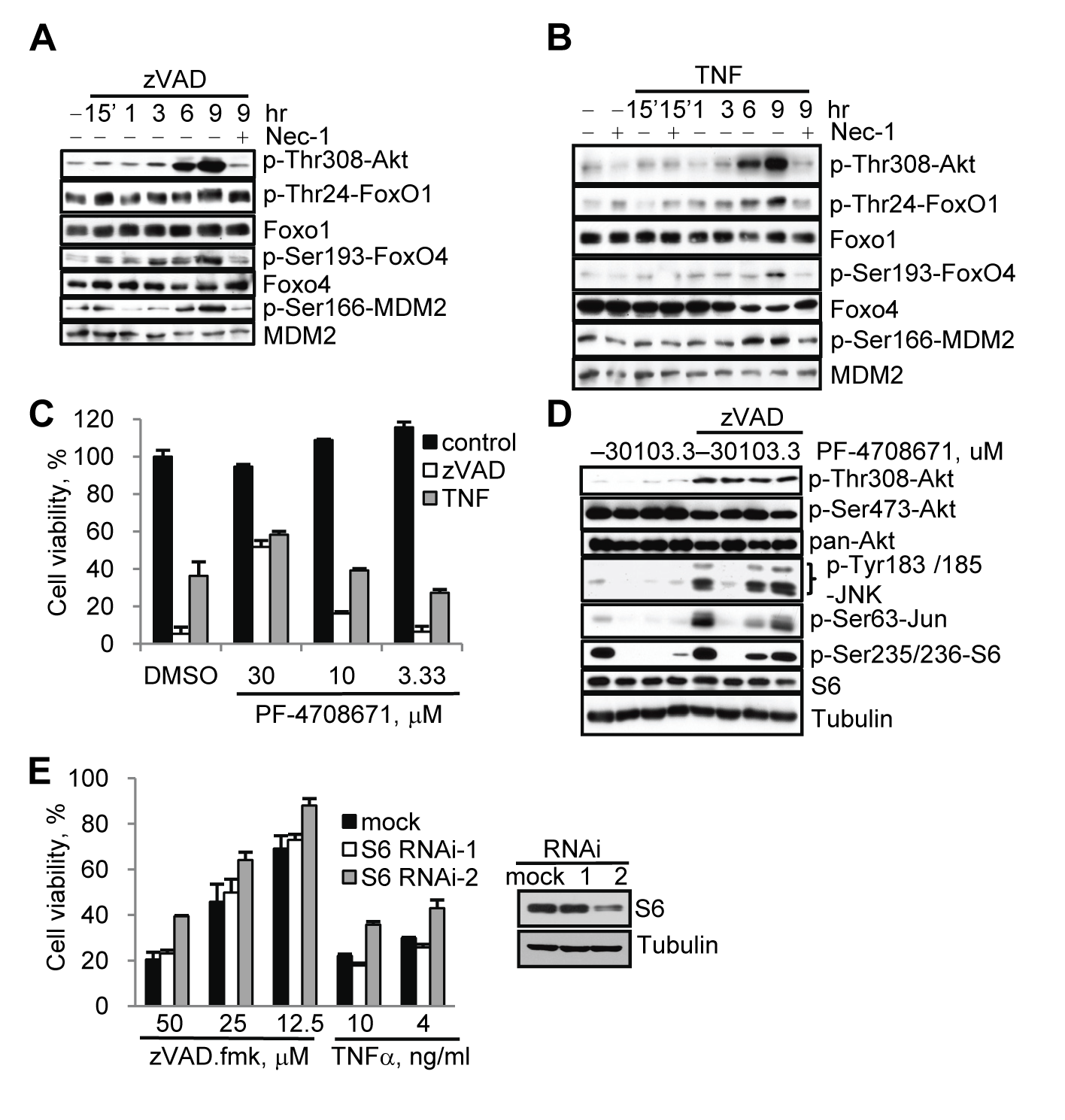

Supplement: Figure S5 — Downstream Akt signaling contributes to the control of necroptosis. (A,B) L929 were stimulated with zVAD.fmk (A) TNFα (B) for the indicated periods of time, followed by western blot using indicated antibodies. (C,D) L929 were stimulated with TNFα or zVAD.fmk in the presence of the indicated concentrations of PF-4706871. Viability was determined after 24 hr (C). Western blot samples were collected after 9 hr (D). (E) L929 cells were transfected with S6 siRNAs. After 48 hr, necroptosis was induced by TNFα and zVAD.fmk for 24 hr. Inset, levels of S6 were determined 48 hr after transfection. In all graphs, average±SD was plotted. (TIF) [file pone.0056576.s005.tif]

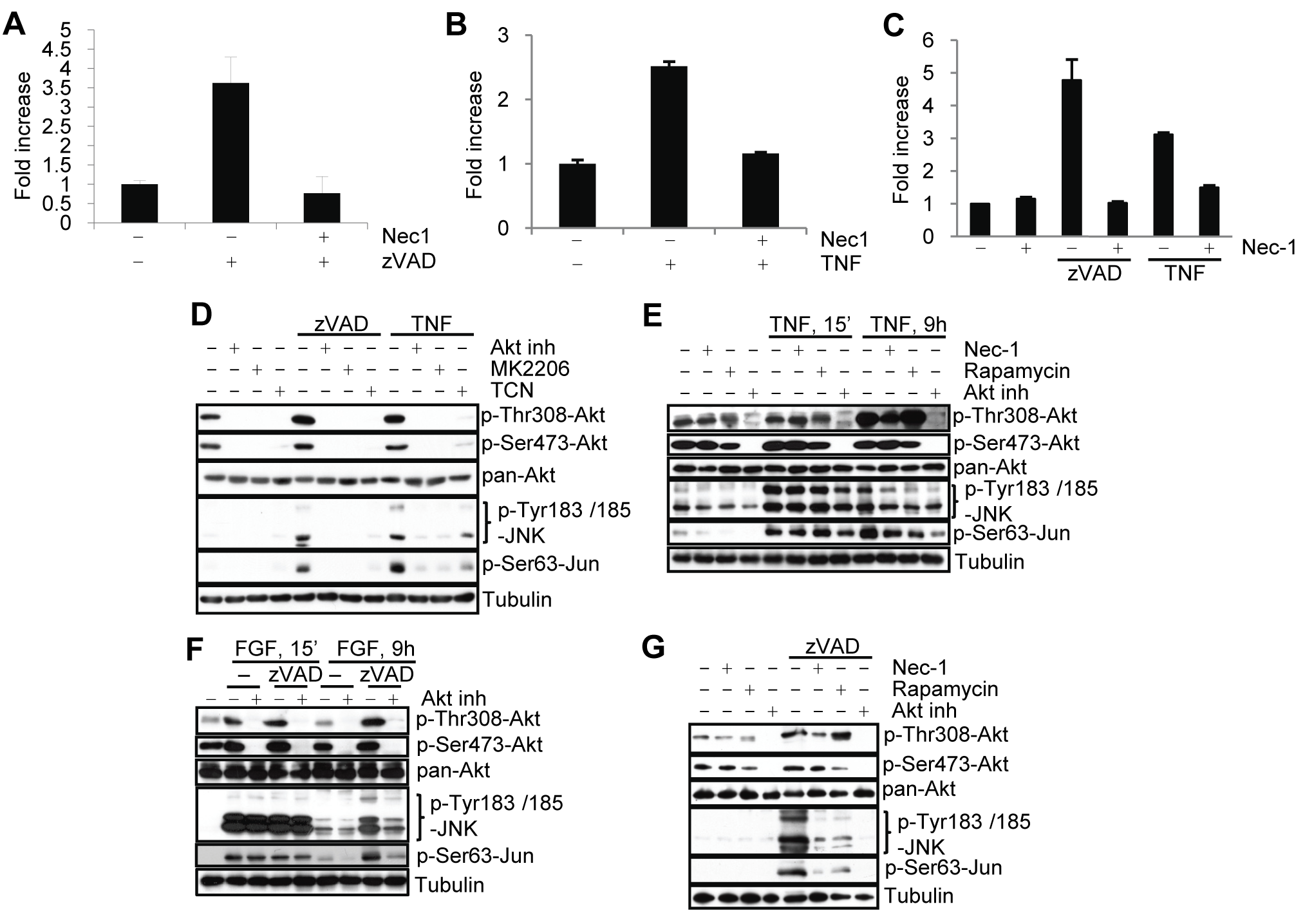

Supplement: Figure S6 — Akt and mTORC1 contribute to autocrine TNFα synthesis and JNK activation during necroptosis. (A,B) L929 cells were stimulated by zVAD.fmk (A) and human TNFα (B) for 9 hr. Cell lysates were subjected to mouse TNFα ELISA. (C) L929 cells were stimulated by zVAD.fmk and TNFα in the presence of either Nec-1 followed by measurement of TNFα mRNA levels by qRT-PCR at 9 hr. (D) L929 cells were stimulated by zVAD.fmk and TNFα in the presence of Akt inh VIII (10 µM), MK2206 (10 µM) and TCN (100 µM) followed by western blot at 9 hrs. (E) Cells were stimulated with TNFα for 15 min or 9 hr in the presence of Nec-1, Akt inh VIII and rapamycin. Western blot samples were collected after 9 hr. (F) L929 cells were stimulated with bFGF and zVAD.fmk (serum free conditions) for 15 min and 9 hr in the presence of Akt inh. VIII and analyzed by western blot. (G) L929 cells were stimulated by zVAD.fmk in the presence of Nec1, Akt inh VIII, or rapamycin followed by western blot at 9 hrs. In all graphs, average±SD was plotted. (TIF) [file pone.0056576.s006.tif]

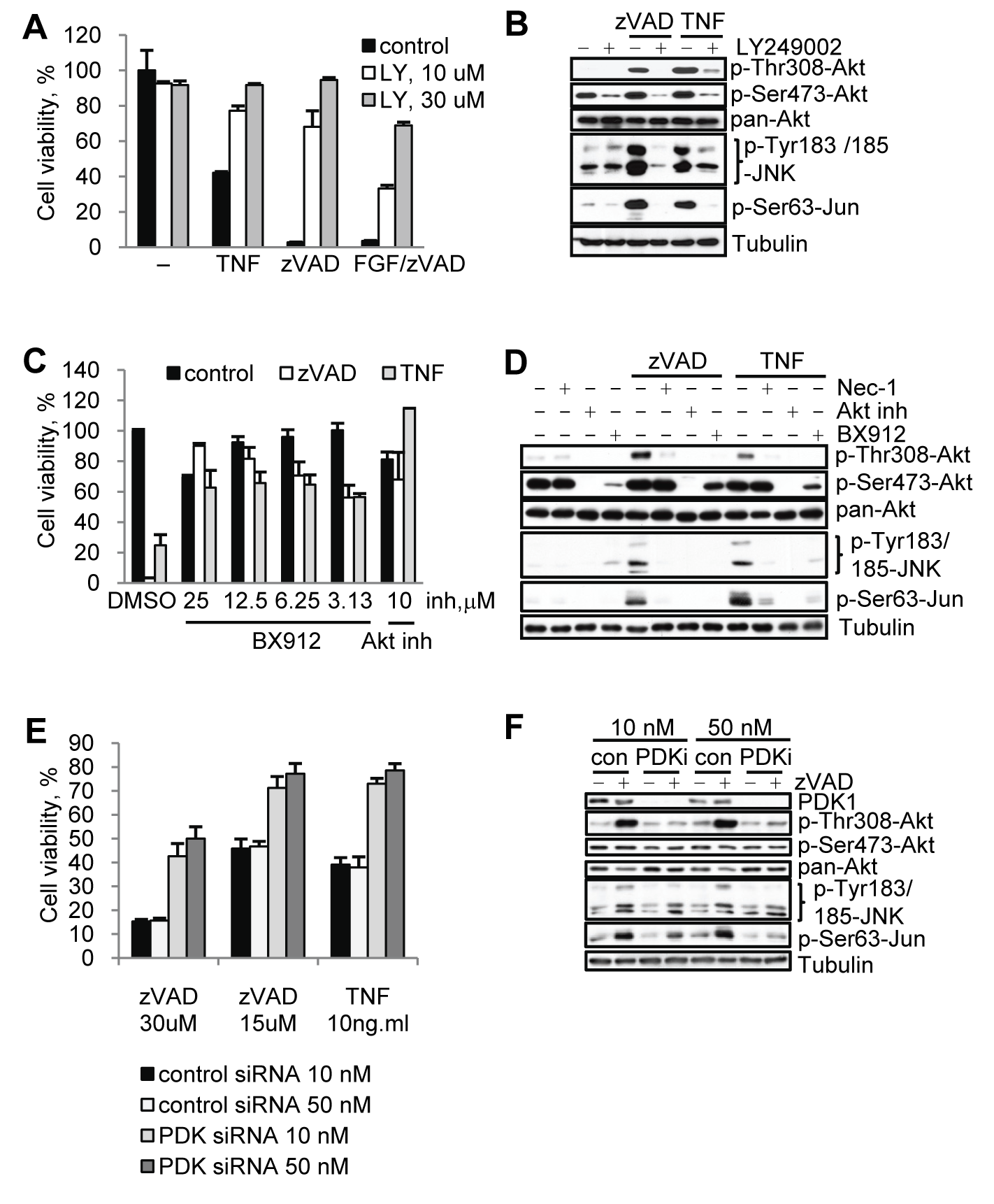

Supplement: Figure S7 — PI3-Kinase and PDK1 mediate the increase in Akt Thr308 phosphorylation under necroptotic conditions. (A-D) L929 cells were stimulated by zVAD.fmk and TNFα in the presence of LY249002 (A,B) or BX912 (C,D). Viability assays were performed after 24 hr (A,C). Western blot samples were collected after 9 hr. (B,D). (E,F) L929 cells were transfected with PDK1 siRNAs. After 72 hr, necroptosis was induced by TNFα or zVAD.fmk. Viability assays were performed after 24 hr (E). Western blot samples were collected after 9 hr (F). In all graphs, average±SD was plotted. (TIF) [file pone.0056576.s007.tif]

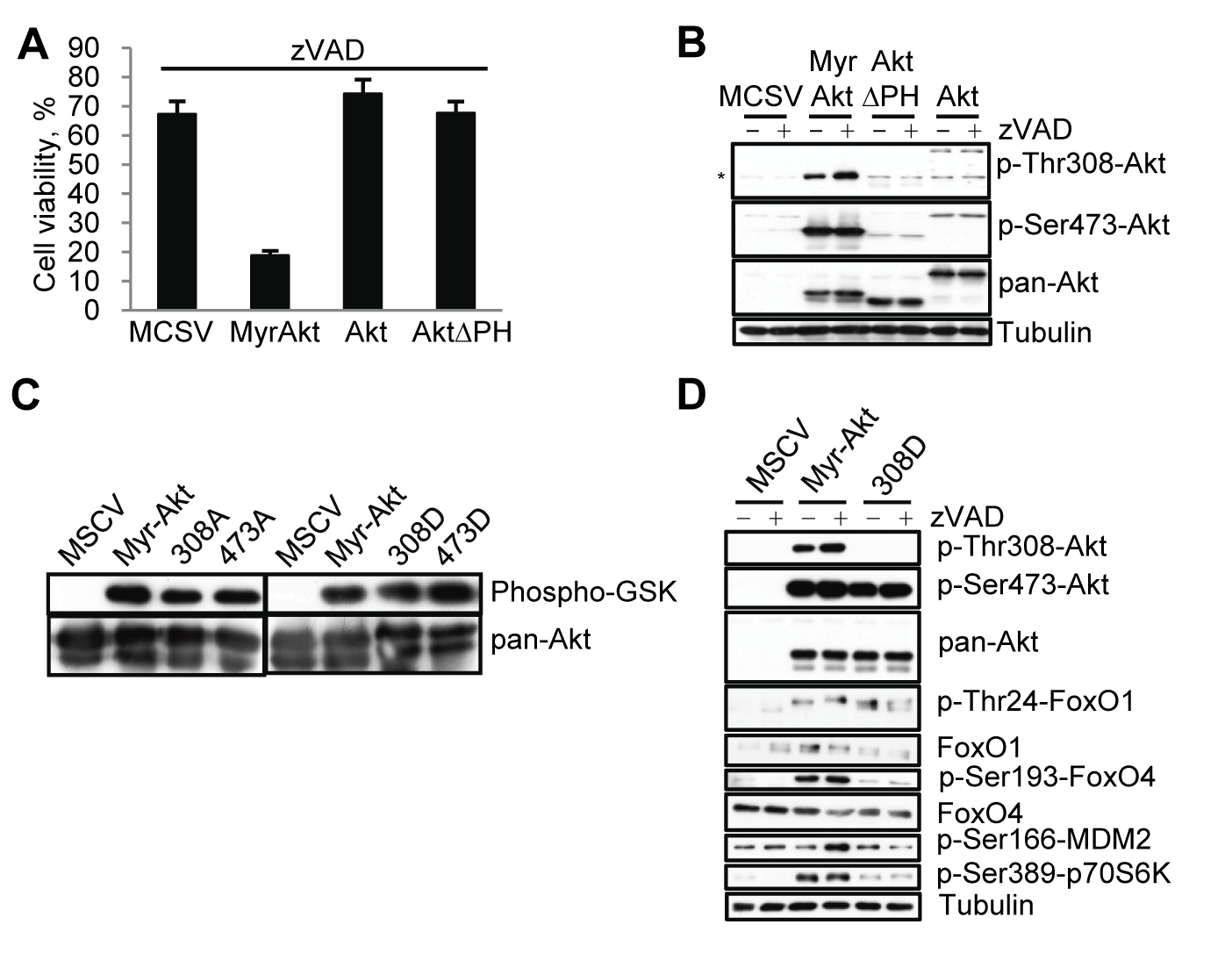

Supplement: Figure S8 — Constitutively active Myr-Akt promotes necroptosis under serum free conditions. (A,B) L929 cells expressing Myr-Akt, full length Akt and a mutant lacking the PH domain were treated with zVAD.fmk under serum free conditions, followed by viability measurement at 24 hr (A) or western blot at 9 hr (B). * - non-specific band, coinciding with the migration of Myr-Akt was detected by some lots of the p308 antibody. (C) L929 cells expressing Myr-Akt and Ala and Asp mutants of Thr308 and Ser473 were immunoprecipitated from L929 cells and their in vitro catalytic activity towards GSK-3β peptide was determined. (D) L929 cells expressing Myr-Akt or the T308D mutant were treated with zVAD.fmk for 9 hrs under serum free conditions followed by western blot analysis. In all graphs, average±SD was plotted. (TIF) [file pone.0056576.s008.tif]

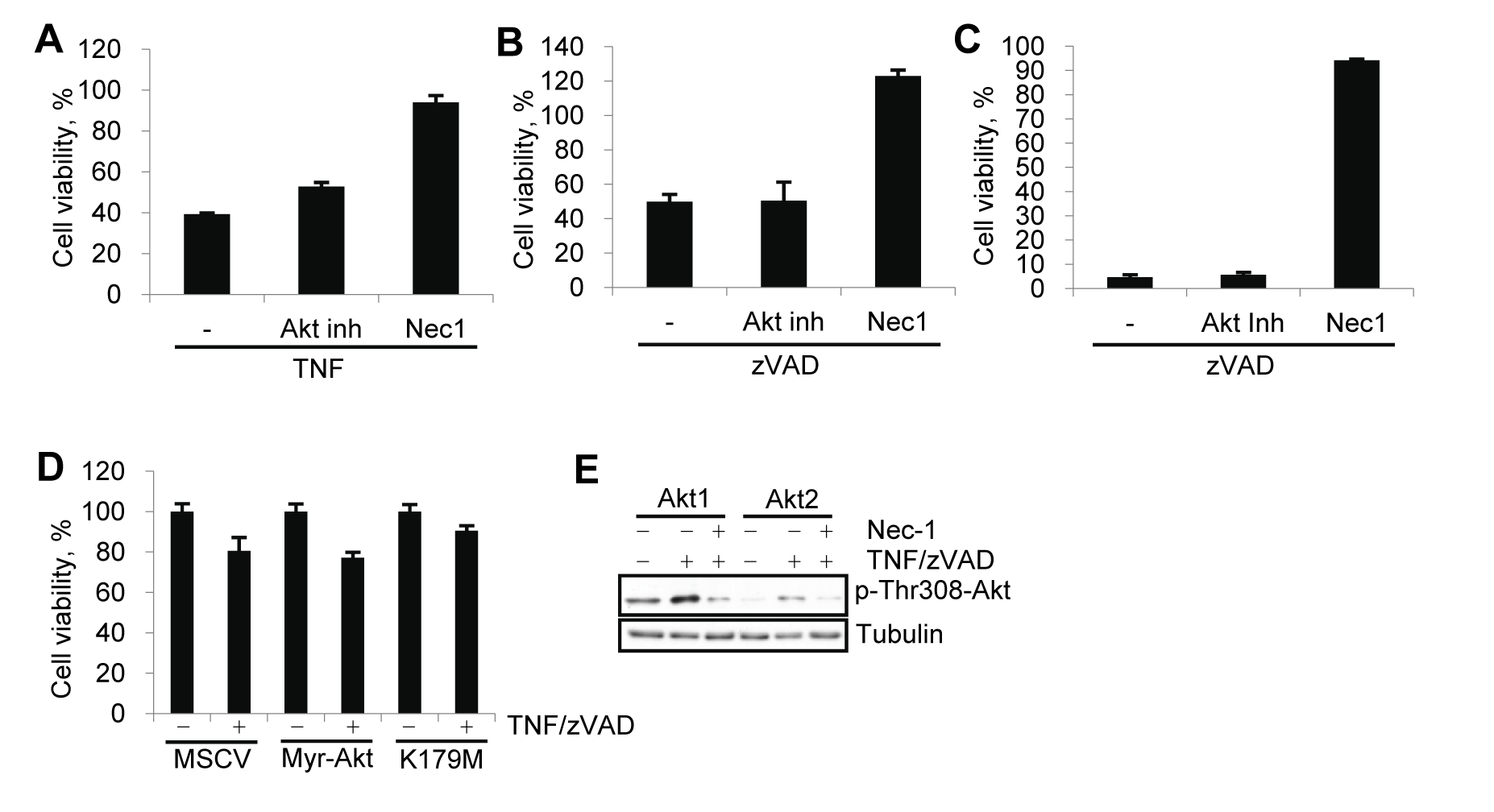

Supplement: Figure S9 — Akt signaling contributes to autocrine TNFα production in multiple cell types. (A) FADD-deficient Jurkat cells were treated with TNFα in the presence of Nec-1 or Akt inh VIII. Cell viability was assayed after 24 hrs. (B,C) RAW 264.7 (B) or J774A.1 (C) were treated with zVAD.fmk (100 uM or 50 uM respectively). Cell viability was assayed after 24 hrs. (D,E) Akt deficient mouse lung fibroblasts stably expressing Myr-Akt or Myr-Akt K179M mutant, were stimulated with TNFα and zVAD.fmk under serum free conditions for 24 hr, followed by cell viability assay or (D) western blot analysis (E). (F) Mouse lung fibroblasts expressing one isoform of Akt (Akt1 or Akt2) were treated with zVAD.fmk and TNFα followed by cell viability assay. In all graphs, average±SD was plotted. (TIF) [file pone.0056576.s009.tif]

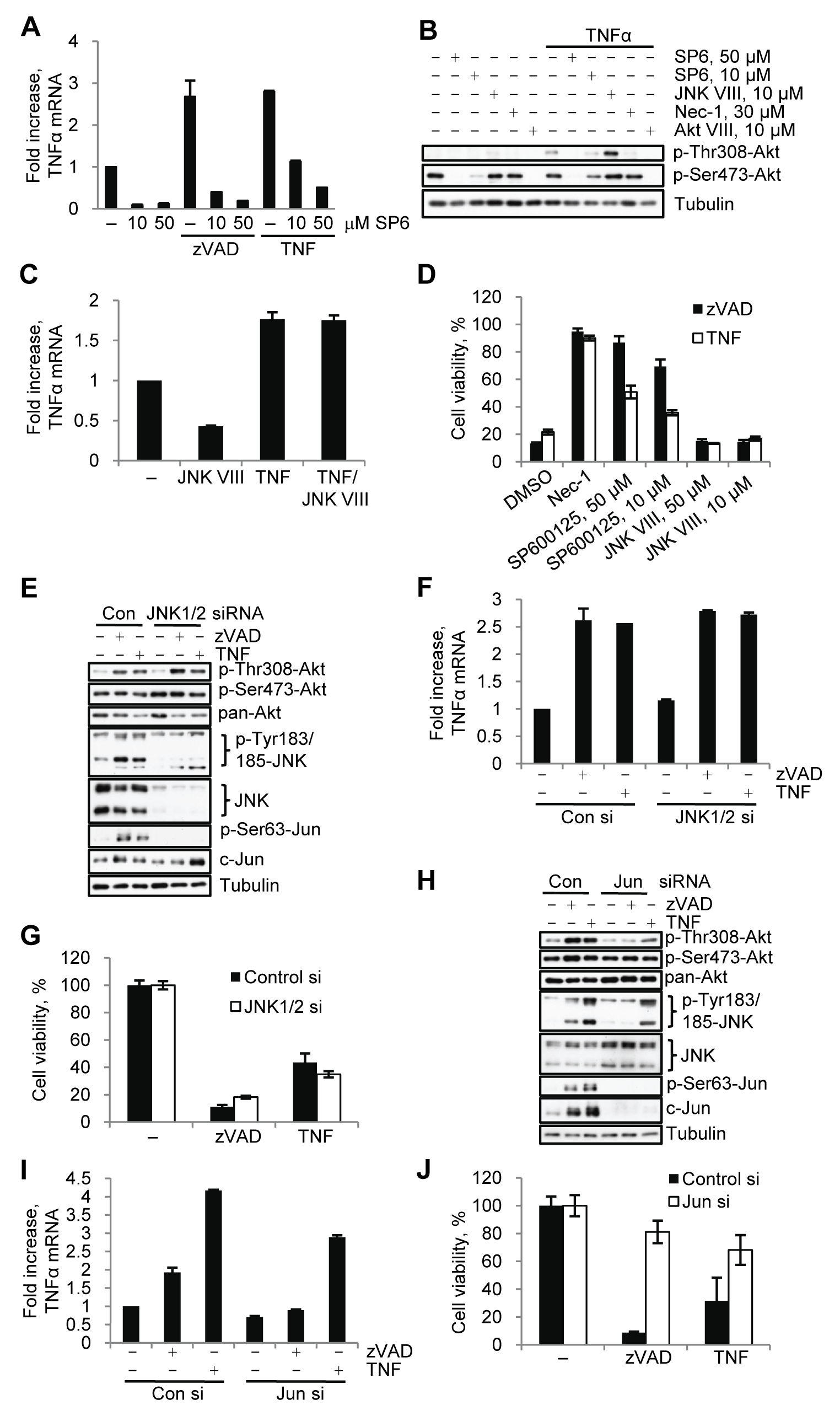

Supplement: Figure S10 — JNK and c-Jun differentially contribute to autocrine TNFα production and cell death. (A) Cells were treated with TNFα or zVAD.fmk with or without SP600125 (SP6) followed by evaluation of TNFα mRNA levels by qRT-PCR at 9 hrs. (B) L929 cells were treated with TNFα for 9 hrs in the presence of the JNK inhibitor SP600125 (SP6), Nec-1, JNK inh. VIII, or Akt inh. VIII and analysed by western blot. (C) Cells were treated with TNFα with or without JNK inhibitor VIII followed by evaluation of TNFα mRNA levels by qRT-PCR at 9 hrs. (D) Cells were treated with zVAD.fmk or TNFα with either SP600125 or JNK inh VIII followed by viability assay at 24 hr. (E-G) L929 cells transfected with JNK1 and JNK2 siRNAs for 72 hrs were treated with zVAD.fmk or TNFα followed by western blot at 9 hr (E), evaluation of TNFα mRNA levels by qRT-PCR at 9 hrs (F), or viability assay at 24 hr (G), (H-J) L929 cells transfected with c-jun siRNAs for 72 hrs were treated with zVAD.fmk or TNFα followed by western blot at 9 hr (H), evaluation of TNFα mRNA levels by qRT-PCR at 9 hrs (I), or viability assay at 24 hr (J), In all graphs, average±SD was plotted. (TIF) [file pone.0056576.s010.tif]
